# Conservation and divergence of ADAM family proteins in the *Xenopus *genome

**DOI:** 10.1186/1471-2148-10-211

**Published:** 2010-07-14

**Authors:** Shuo Wei, Charles A Whittaker, Guofeng Xu, Lance C Bridges, Anoop Shah, Judith M White, Douglas W DeSimone

**Affiliations:** 1 Department of Cell Biology and the Morphogenesis and Regenerative Medicine Institute, University of Virginia, Charlottesville, VA 22908, USA; 2 David H. Koch Institute for Integrative Cancer Research at Massachusetts Institute of Technology, Cambridge, MA 02139, USA; 3 Department of Chemistry, University of Central Arkansas, Conway, AR 72035, USA

## Abstract

**Background:**

Members of the disintegrin metalloproteinase (ADAM) family play important roles in cellular and developmental processes through their functions as proteases and/or binding partners for other proteins. The amphibian *Xenopus *has long been used as a model for early vertebrate development, but genome-wide analyses for large gene families were not possible until the recent completion of the *X. tropicalis *genome sequence and the availability of large scale expression sequence tag (EST) databases. In this study we carried out a systematic analysis of the *X. tropicalis *genome and uncovered several interesting features of ADAM genes in this species.

**Results:**

Based on the *X. tropicalis *genome sequence and EST databases, we identified *Xenopus *orthologues of mammalian ADAMs and obtained full-length cDNA clones for these genes. The deduced protein sequences, synteny and exon-intron boundaries are conserved between most human and *X. tropicalis *orthologues. The alternative splicing patterns of certain *Xenopus *ADAM genes, such as *adams 22 *and *28*, are similar to those of their mammalian orthologues. However, we were unable to identify an orthologue for ADAM7 or 8. The *Xenopus *orthologue of ADAM15, an active metalloproteinase in mammals, does not contain the conserved zinc-binding motif and is hence considered proteolytically inactive. We also found evidence for gain of ADAM genes in *Xenopus *as compared to other species. There is a homologue of ADAM10 in *Xenopus *that is missing in most mammals. Furthermore, a single scaffold of *X. tropicalis *genome contains four genes encoding ADAM28 homologues, suggesting genome duplication in this region.

**Conclusions:**

Our genome-wide analysis of ADAM genes in *X. tropicalis *revealed both conservation and evolutionary divergence of these genes in this amphibian species. On the one hand, all ADAMs implicated in normal development and health in other species are conserved in *X. tropicalis*. On the other hand, some ADAM genes and ADAM protease activities are absent, while other novel ADAM proteins in this species are predicted by this study. The conservation and unique divergence of ADAM genes in *Xenopus *probably reflect the particular selective pressures these amphibian species faced during evolution.

## Background

ADAMs belong to the M12B subfamily of metalloproteinases and metalloproteinase-like proteins [1]. A prototype ADAM is a type I transmembrane protein, but some ADAMs are also present as soluble forms, either due to alternative splicing or protease-mediated cleavage ("shedding") from the cell surface [2,3]. ADAMs are multi-domain proteins with an extracellular metalloproteinase domain, a disintegrin domain and a cysteine-rich domain; therefore they are also called MDC (metalloproteinase/disintegrin/cysteine-rich) proteins. Some but not all ADAMs contain a canonical zinc-binding motif within the metalloproteinase domain, which is required for protease activity [2,3]. The disintegrin domain can selectively interact with different integrins [4]; together with the cysteine-rich domain, it may modulate cell-cell and cell-matrix adhesion [4-6], as well as substrate recognition by the metalloproteinase domain [7,8]. The cytoplasmic tail contains binding sites for a variety of cellular proteins, and may be involved in inside-out signaling that regulates the activity of the ectodomain [9-11].

A phylogenetic tree of ADAMs identified in different species can be found in the tree families database TreeFam [12]. About half of the ADAMs are solely or predominantly expressed in the testis of mammals (i.e. testis-specific), with no orthologue found in nonmammalian species [12,13]. Functions of these ADAMs are thus mainly related to mammalian reproduction. The other ADAMs are expressed widely in tissues and organs other than the testis. Many of these ADAMs are implicated in embryonic development. Mice lacking or carrying defective ADAM genes often display developmental abnormalities that vary from defects in adipogenesis and myogenesis [14] and mild dysfuctions in the central nervous system (CNS)[15,16], to more severe defects such as early embryonic and perinatal lethality [17-19]. The activities of ADAMs are also linked to a variety of human diseases, such as cancer [20] and cardiovascular diseases [21], as well as rheumatoid arthritis and other inflammatory diseases [22]. However, the molecular bases for the functions of ADAMs are largely unappreciated, and identifying the *in vivo *substrates and binding partners for ADAMs remains a major challenge [2,23]. Furthermore, functional redundancy between two or more ADAMs seems to be a common phenomenon [24,25], posing an additional difficulty for understanding the roles of ADAMs in development and diseases.

One area that has drawn increasing interest concerns the roles of ADAMs in cell signaling, mainly via shedding of cell surface proteins by ADAM metalloproteinase activities. Through this process, ADAMs release key extracellular signaling proteins (e.g. growth factors such as EGF and cytokines such as TNF-α) from the cell surface [3,22]. ADAMs also participate in the generation of key intracellular signals (e.g. Notch and ErbB4 intracellular domain) by performing an obligate ectodomain cleavage event prior to a regulated intramembraneous proteolytic cleavage [26,27]. Recently, a subgroup of nonproteolytic ADAMs, ADAMs 11, 22 and 23, were shown to function as receptors for the secreted Leucine-rich glioma inactivated (LGI) proteins, which are required for proper synaptic transmission in the brain [28,29]. Regulation of cell adhesion and migration is another major function of ADAMs. A large body of evidence suggests that ADAM disintegrin domains can interact with integrins, although most ADAMs do not contain the conserved RGD integrin binding sequence [30,31]. The cysteine-rich domain of ADAM12 can also mediate cell adhesion by binding cell surface syndecans [6,32]. Furthermore, ADAM metalloproteinase activities are also involved in remodeling extracellular matrix and regulating cell-cell adhesion through cleavage of extracellular matrix components (e.g. type IV collagen and fibronectin) [33-35] and adhesion molecules (e.g. cadherins) [36-40].

The amphibian *Xenopus laevis *has been used for decades as a model system for studying early vertebrate development. The easy availability of large numbers of fertilized eggs, the ability of the embryos to develop and be manipulated *in vitro*, and the transparent epidermis of the tadpoles provide some practical advantages over mammalian and other models. However, the pseudo-tetraploid genome and long generation time of this species prevented it from being widely used for genetic and genomic research. These difficulties were overcome in large part by the adoption of *Xenopus tropicalis*, a closely related species with a true diploid genome and a relatively short generation time. The genome sequence of *X. tropicalis *is now complete, and large collections of EST clones from both *X. tropicalis *and *X. laevis *are available, providing useful tools for systematic prediction and characterization of large families of genes. A recently published genome-wide analysis of the matrix metalloproteinases (MMPs), another family of extracellular metalloproteinases that is related to the ADAMs, revealed evolutionary conservation and distinct duplication patterns of MMPs in *X. tropicalis *as compared to humans and other mammals [41]. Several ADAM genes have been cloned previously from *X. laevis *and characterized for their functions in fertilization and development [34,42-45]. Given the key roles of these ADAMs *in vivo*, it is important to establish their orthologues in mammals and to identify their paralogues in the *Xenopus *genome.

As an initial step for systematic studies of all ADAM family proteins in the frog species, we started out to identify the homologues of known vertebrate ADAMs that are expressed in *X. tropicalis*. Our study shows that most non-testis specific ADAMs are conserved between frogs and mammals. However, some potential loss or gain of ADAM genes (and possibly functions) in *X. tropicalis *as compared to mammals suggests evolutionary divergence of ADAMs in this amphibian species.

## Results and discussion

### ADAM genes in the X. tropicalis genome

Searches of the current version (v4.1) of the *X. tropicalis *genome assembly yielded homologues for most known vertebrate non-testis specific ADAMs. Sequences of these predicted ADAM genes were then used to search against the EST databases (or to design primers for experimental cloning) to obtain full-length cDNA clones from *X. tropicalis *and, where possible, *X. laevis*. No orthologues of the mammalian testis-specific ADAMs have been found in any nonmammalian species [12,13]; similarly, we were unable to identify a *Xenopus *orthologue for any of these ADAMs. A predicted gene encoding the orthologue of *X. laevis *ADAM16 (previously known as xMDC16), an ADAM expressed only in testis with a potential role in fertilization [42], was localized to Scaffold_40 of *X. tropicalis *genome. No orthologue of ADAM16 was found in any other species examined, including mammals, fish and chicken. Therefore it is likely that ADAM16 function is limited to reproduction of the frogs and related species.

**Figure 1:**
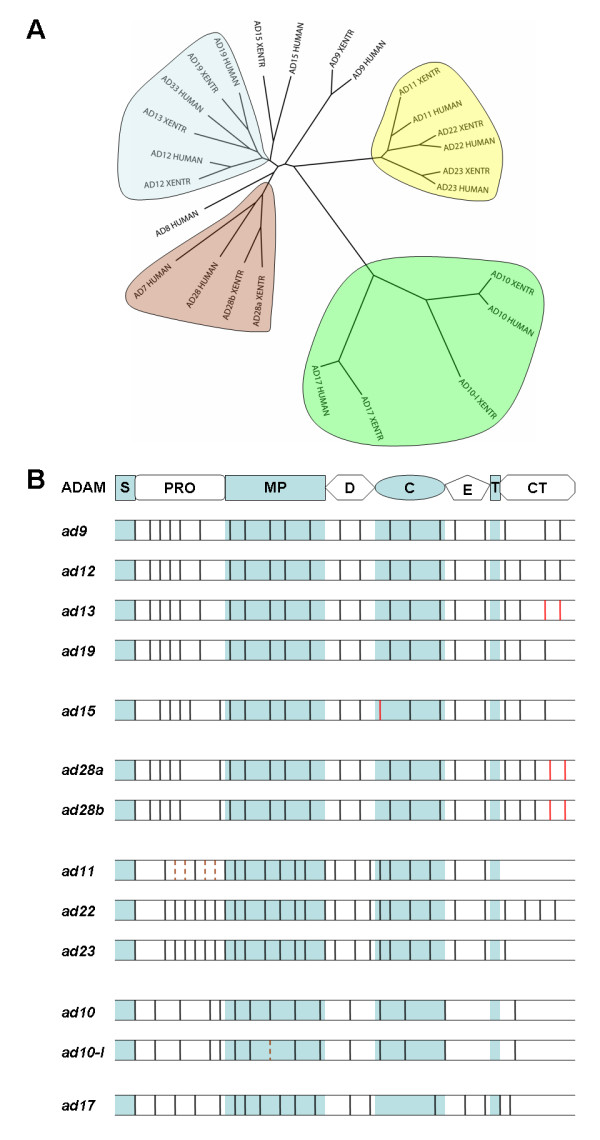
**Phylogenetic and splicing analyses of human and *X. tropicalis *ADAMs**. A) Phylogenetic tree of human and *X. tropicalis *ADAMs. Protein sequences of human and *X. tropicalis *(XENTR) ADAMs were aligned, and a neighbor-joining tree was drawn using ClustalX. Clades are highlighted by different colors. See Additional File 1 for an expanded phylogenetic tree including other representative vertebrate species, and Additional File 6, A-C for trees generated using alternative models. B) Comparison of splicing patterns of human and *X. tropicalis adam *transcripts. Splice sites within different domains (as indicated at top) of *X. tropicalis adam *genes, as compared with their human orthologues, are shown. Human *adams 33 *and *10 *were used in the comparison with *X. tropicalis adams 13 and 10-like *(*ad10-l*), respectively. Black vertical lines represent splice sites that are conserved between these two species, and solid and dotted red vertical lines represent splice sites that are used by *X. tropicalis adams *or their human orthologues, respectively, but not both. *Adam *genes are divided into different subgroups (*adams 9/12/13/19*, *15*, *28a/b*, *11/22/23*, *10/10-l *and *17*) based on their splicing patterns, as in Ref. 46. S, signal peptide; PRO, propeptide; MP, metalloproteinase domain; D, disintegrin domain; C, cysteine-rich domain; E, EGF-like domain; T, transmembrane region; CT, cytoplasmic tail.

A phylogenetic tree of *X. tropicalis *and human non-testis specific ADAMs is shown in Figure 1A, in which these ADAMs are divided into several subfamilies ("clades"). Results of this analysis are consistent with an expanded tree including other representative vertebrate species (Additional File 1), and with previously published phylogenetic studies of ADAMs from various species [12,30,46]. Comparison of the exon-intron structures of *X. tropicalis *ADAM genes with those of human ADAM genes [46] reveals conservation of most of the splice sites between orthologues (Figure 1B). ADAMs can also be divided into clades based on their splicing patterns, as suggested by Kleino et al. ([46] and Figure 1B). While this subgrouping method places emphasis on conservation and divergence in gene structure, the method used in Figure 1A is based on sequence similarity and probably better reflects the conservation and evolution in gene function. In general the phylogeny-based subgrouping (Figure 1A) agrees well with the splicing-based method ([46] and Figure 1B), but it also differs from the latter in two aspects: the exclusion of ADAM9 from the meltrin clade (ADAMs 12, 13/33 and 19) and the inclusion of ADAMs 10 and 17 into the same clade. We believe that the former (Figure 1A) better indicates the functional relationship among ADAM paralogues (as discussed in the following sections). The phylogeny-based subgrouping is the method of choice here because sequence homology and gene functions are the primary focus of this study.

Sequence information and expression patterns of identified *Xenopus *ADAM genes are summarized in Table 1. Several ADAMs, including ADAMs 9, 10, 11, 12, 17, 19, 22 and 23, are known to be required for normal development and health in mice [14-19,47-50]. As expected, all of these essential ADAMs are conserved in frogs, with >50% identities in protein sequence between *X. tropicalis *and human orthologues; the other ADAMs are more divergent (Table 1). In cases where full-length sequence information of *X. laevis *cDNA is also available, the similarities between *X. tropicalis *and *X. laevis *orthologues are high (>80% identities in protein sequence), consistent with the close relationship between these two species (Table 1).

**Table 1:** *X. tropicalis *ADAM genes identified in this study

Encoded ADAM	GenBank accession	% identity with closest human homologue	% identity with *X. laevis *orthologue	Expression pattern*
ADAM9	HM467820	69	91	Widespread

ADAM10	DQ287908	82	93	Widespread

ADAM10-like	HM483366	43 (with ADAM10)	ND	Widespread

ADAM11^†^	HM467821	76	95	Tailbud stage head; tadpole; metamorphic tail; adult brain (high level)

ADAM12	DQ393787	66	ND	Gastrula; neurula; tadpole

ADAM13/33	DQ393788	53 (with ADAM33)	91	Gastrula; neurula; tadpole; metamorphic tail; adult spleen

ADAM15^†^	HM467822	44	81	Gastrula; metamorphic limb; adult brain, lung, testis

ADAM17	HM483367	68	91	Neurula; adult brain, testis, oviduct

ADAM19	DQ393789	59	85	Gastrula; neurula; tadpole

ADAM22^†Δ^	HM467823 HM467824 HM467825	71	83	Tadpole; metamorphic brain/spinal cord; adult brain (high level), testis

ADAM23^†^	HM467826	72	ND	Tadpole; adult brain (high level), spleen, kidney, thymus

ADAM28a^‡^	HM467827	47	82	Widespread

ADAM28b^‡^	HM467828	44	ND	Neurula; tadpole; adult spleen, bone, kidney

* Expression patterns were estimated by the presence of ESTs (full or partial) in different organs and/or developmental stages, as deposited in GenBank. "Widespread" was assigned when ESTs are present in multiple (>20) organs, and "high level" was assigned when multiple (>20) EST entries are present in a single organ at a single developmental stage and account for >50% of all ESTs found in GenBank.

^† ^ADAMs predicted to be proteolytically inactive.

^‡ ^Two additional *adam28 *homologues (*adam28c *and *d*) were identified on Scaffold 30, but full-length cDNA clones have not been obtained.

^Δ ^Three alternatively spliced isoforms of *X. tropicalis *ADAM22 were identified in this study. ISO1 (HM467823) was used to calculate sequence identities here.

ND: not determined.

**Figure 2:**
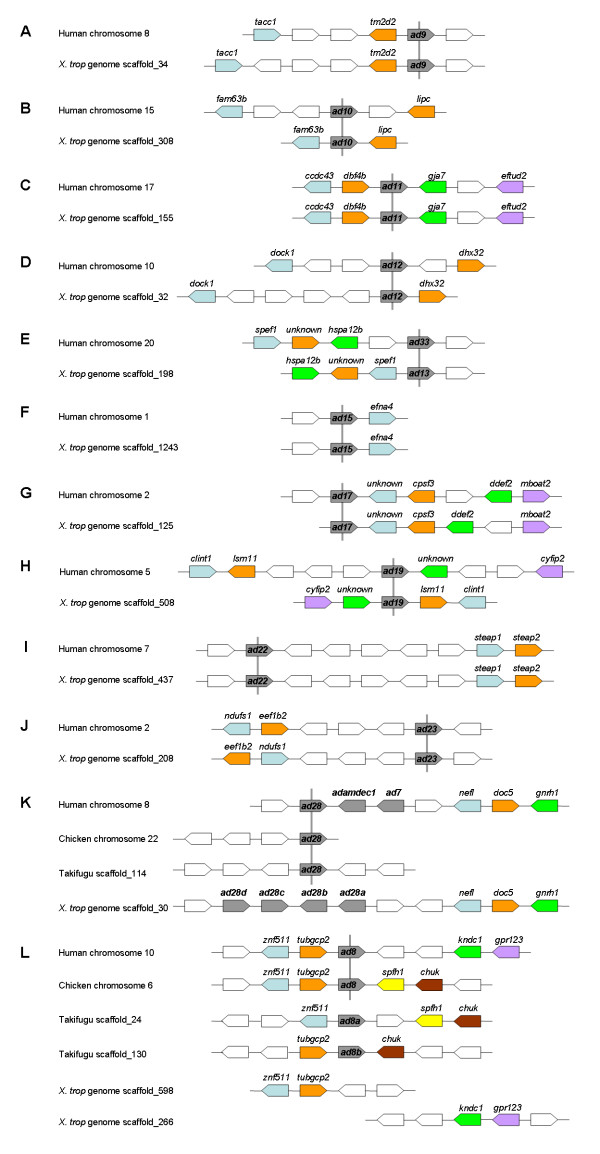
**Synteny analyses of human and *X. tropicalis *ADAM genes**. A-J) Synteny of most ADAM genes is conserved between human and *X. tropicalis*. K) The *adam7/28/adamdec1 *gene cluster in human is replaced by four tandem *adam28 *homologues in the *X. tropicalis *genome. L) The syntenic region surrounding *adam8 *in human separates into two fragments in the *X. tropicalis *genome, and *adam8 *seems to be lost. Chicken and fish (takifugu) genes are included in K and L for comparison, and results obtained from medaka and stickleback are similar to takifugu (not shown). ADAM and ADAMDEC1 genes are shown in grey, and orthologues are connected with a vertical line. Orthologues of other genes are shown in the same colors, and genes whose positions are not conserved in the species examined are shown in white.

The orthologous relationship between human and *X. tropicalis *ADAMs is further supported by synteny analyses, which show the conservation of genetic linkages between most *adams *and nearby genes in these two species (Figure 2). However, we were unable to identify any likely orthologue for ADAM7 or 8 in the *X. tropicalis *genome or in *X. tropicalis*/*X. laevis *EST databases. ADAM7 is mainly expressed in the testis [51], but limited expression is also detected in muscles and kidney [52]. This ADAM seems to be mammal-specific and no orthologue has been identified to date in other animals [12]. Therefore it is perhaps not surprising that it is also absent in frogs. In the human genome *adam7 *lies next to *adam28 *and *adamdec1*, whereas in *X. tropicalis *this metalloproteinase gene "cluster" is replaced by four homologues of *adam28 *in tandem (Figure 2K; see further discussion below). ADAM8 is one of the most widely studied ADAMs, with potential roles in cancer and inflammatory diseases [53]. However, mice with targeted deletion of the ADAM8 gene do not display any apparent developmental or pathological phenotypes [54], indicating that this gene is dispensable for normal development and health. The human *adam8 *gene was mapped to chromosome 10, with *tubgcp2 *and *znf511 *on one side and *kndc1 *and *gpr123 *on the other side (Figure 2L). Although the loci of *adam8*, *tubgcp2 *and *znf511 *are also conserved in the chicken genome, *kndc1 *and *gpr123 *genes are not localized in the vicinity; instead two other genes, *spfh1 *and *chuk*, are found in this region (Figure 2L). There are two *adam8 *homologues in fish (takifugu), one flanked by *znf511 *and *spfh1/chuk*, and the other by *tubgcp2 *and an additional homologue of *chuk *(Figure 2L), suggesting possible genome duplication associated with gene loss in this region. In the *X. tropicalis *genome, *tubgcp2 *and *znf511 *were localized to scaffold_598, whereas *kndc1 *and *gpr123 *were localized to a different scaffold, scaffold_266 (Figure 2L), and *spfh1 *and *chuk *lie separately on two other scaffolds (not shown). No *adam8 *homologue was found on any of these scaffolds. These results suggest that this genomic region has undergone extensive rearrangement during evolution, and that the *adam8 *gene was probably lost in the frogs. The possible absence of an ADAM8 orthologue in the *X. tropicalis *genome may reflect a poor conservation of this ADAM metalloproteinase during evolution. We also cannot rule out the possibility that the available genome sequence data may be incomplete.

In addition to potential loss of ADAM genes as compared to the mammalian genomes, there is also evidence for additional ADAM genes in the *X. tropicalis *genome. A homologue of ADAM10, which we named ADAM10-like, was found in *Xenopus *but absent in most placental mammals. We also identified four homologues of ADAM28 on Scaffold_30 of the *X. tropicalis *genome assembly. Features of *X. tropicalis *ADAMs identified in this study are discussed in the following sections in terms of different ADAM clades as indicated in Figure 1A.

### ADAMs 12, 13 and 19

We previously identified *X. laevis *ADAM13 as a proteolytically active ADAM expressed predominantly in cranial neural crest (CNC) cells [44], an embryonic cell population that gives rise to craniofacial structures in vertebrates [55,56]. Ectopic expression of a protease-dead mutant of ADAM13, as well as antisense morpholino mediated knockdown of ADAM13, inhibits CNC migration in neurula stage embryos [34,40]. A closely related ADAM, ADAM19, was recently found to be required for CNC induction and migration in *X. laevis *[45]. The similarity between the *in vivo *functions of ADAMs 13 and 19 raises the possibility that these two ADAM metalloproteinases may act through similar mechanisms (e.g. by cleaving the same physiologically relevant substrates). Furthermore, there may be additional paralogues with similar roles in CNC development encoded by the frog genome. To address these questions we performed a search for closely related homologues of *X. laevis *ADAMs 13 and 19 in *X. tropicalis*.

**Figure 3:**
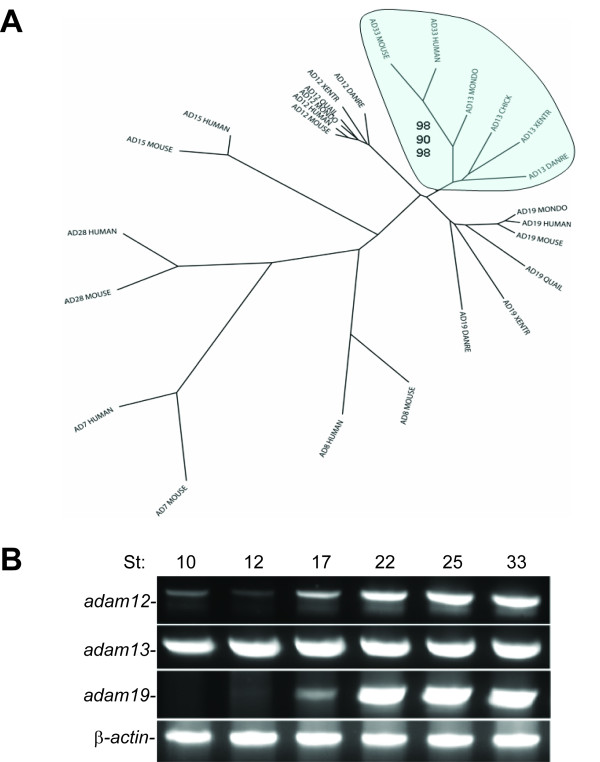
**Sequence and expression analyses of ADAMs 12, 13/33, and 19**. A) Phylogenetic analysis of selected ADAM family members including gene models from opossum and fish. A subset of the ADAM family from human, mouse, opossum (MONDO), bird (QUAIL, CHICK), *X. tropicalis*, and zebrafish (DANRE) was aligned and a neighbor-joining tree was drawn using ClustalX. The ADAM13/33 clade is highlighted, and numbers are bootstrap values (in percentage) from Neighbor (top), Protpars (middle), and Proml (bottom) analyses (see Additional File 6, D-F for trees generated using these models). B) Developmental expression of *adams 12*, *13*, and *19*. RNA from *X. tropicalis *embryos at the indicated stages was analyzed for the presence of *adam12*, *13*, and *19 *transcripts by RT-PCR. Primer pairs for all *adams *were designed to span at least one large intron, so that the PCR products will not be contaminated by amplification of genomic DNA. PCR products for all 3 *adams *were confirmed by DNA sequencing.

Analysis of the *X. tropicalis *genome indicates that there are three ADAMs closely related to *X. laevis *ADAM13: *X. tropicalis *ADAMs 12, 13 and 19. We obtained full-length cDNA constructs for these ADAMs by direct cloning. As shown in Figure 3A, sequence comparison with other ADAM family members suggests that these three proteins constitute an ADAM subfamily with clear orthologous relationships between *Xenopus *and mammalian ADAMs 12 and 19. Although the relationship is not as clear, the most parsimonious explanation is that *Xenopus *ADAM13 and mammalian ADAM33 are orthologues. This is further supported by the synteny analysis, which shows a conserved genetic linkage between *adam13/33 *and *spef1*, *hspa12b *and an unknown gene (Figure 2E). ADAM12, 13/33, and 19 orthologs are detectable in zebrafish, suggesting that this ADAM subfamily was present in the ancestral vertebrate (Figure 3A). RT-PCR analyses showed that ADAMs 12, 13 and 19 are each expressed by neurula stage in *X. tropicalis *(Figure 3B; PCR products were confirmed by DNA sequencing). Functions of these ADAMs in CNC and neural development will be discussed separately (Wei et al., manuscript submitted).

### ADAM15

Human ADAM15 was originally named metargidin (metalloprotease-RGD-disintegrin protein), because it was the first cellular disintegrin protein (and to date the only human ADAM) found to contain the consensus RGD integrin binding site in the disintegrin domain [57]. However, this RGD sequence is not conserved in mouse ADAM15 [58]. The mammalian ADAM15 gene encodes an active metalloproteinase, and several substrates have been identified for this enzyme [59-61]. Mice lacking ADAM15 do not show any apparent deficiencies, but they are more resistant to pathological neovascularization [62].

**Figure 4:**
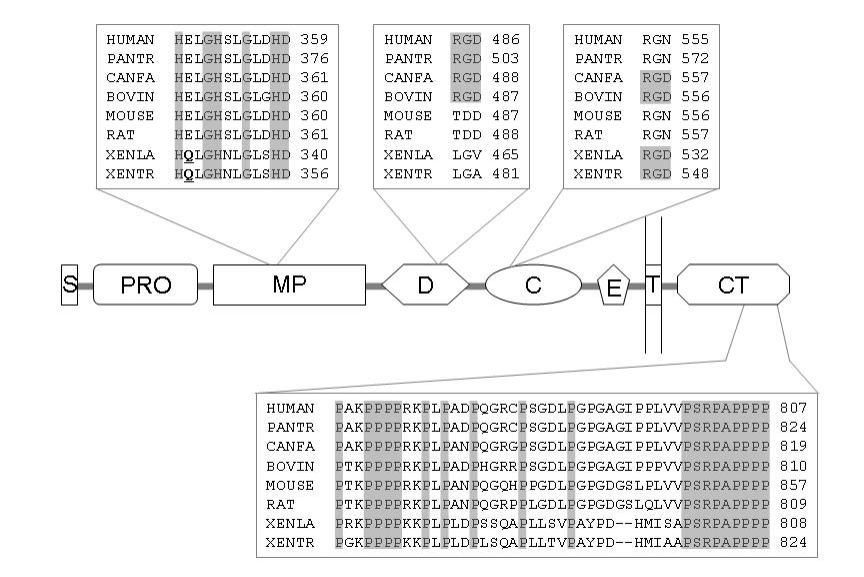
**Sequence comparison of mammalian and *Xenopus *ADAM15**. Sequence alignment of human, chimpanzee (PANTR), canine (CANFA), bovine (BOVIN), mouse, rat, *X. laevis *(XENLA) and *X. tropicalis *ADAM15 was generated using ClustalX. Domain organization of ADAM15 is shown with insets displaying alignments of the zinc-binding motif (the Gln in *Xenopus *ADAM15 is highlighted in bold and underscored), the RGD integrin-binding sites, and the conserved proline residues in the cytoplasmic tail. Conserved residues are highlighted in grey. See Figure 1 legend for abbreviations of domain names and Additional File 2 for a complete sequence alignment that also includes zebrafish ADAM15 proteins.

We searched the *X. tropicalis *genome and EST databases and identified an ADAM15 homologue that is 44% identical to the human protein. Similar to human *adam15 *[46], the gene encoding this homologue was localized next to *efna4 *in the *X. tropicalis *genome (Figure 2F), suggesting that it is the orthologue of ADAM15. A cDNA clone encoding the *X. laevis *orthologue of this protein was also found in the EST databases (BC146626). Surprisingly, unlike the mammalian ADAM15 proteins, which contain the consensus zinc-binding motif HEXGH in the catalytic domain, both *X. tropicalis *ADAM15 and its *X. laevis *orthologue have the sequence HQXGH in this position (Figure 4). An E to Q mutation in the same motif has been shown to result in loss of proteolytic activity in ADAM12 [63], hence it is likely that frogs do not have an active ADAM15 metalloproteinase. Mammalian ADAM15 contains a proline-rich cytoplasmic tail with several potential Src homology-3 (SH3) domain binding sites [64]. As shown in Figure 4, many of these prolines are conserved in mammals and frogs. In contrast, while the mammalian ADAM15 proteins share a strikingly similar signal peptide, this peptide is less conserved in *Xenopus *ADAM15 (Additional File 2). Finally, primate, canine and bovine ADAM15 proteins have a consensus RGD integrin binding site in the disintegrin domain; this sequence is not conserved in rodent or frog ADAM15. Instead *Xenopus *ADAM15 proteins contain an RGD sequence within the cysteine-rich domain (Figure 4). Interestingly, this second RGD sequence is also present in canine and bovine ADAM15, whereas in the primate and rodent orthologues it is replaced by the sequence RGN (Figure 4). A possible explanation is that the ancestor of vertebrate ADAM15 might have two RGD integrin binding sites, one in the disintegrin domain and the other in the cysteine-rich domain. Both of these RGD sequences were maintained in the canine and bovine lineages (both belong to Laurasiatheria) but lost in rodents, while primates and frogs each retained a different RGD sequence. The conservation of the synteny, the SH3 binding motifs and the RGD sequences indicates that these *Xenopus *homologues are real orthologues of mammalian ADAM15, although the metalloproteinase consensus sequence was lost during evolution. In contrast, the two zebrafish ADAM15 homologues both contain the conserved zinc-binding motif but lack either RGD site (Additional File 2).

### ADAM28

ADAM28 (also known as MDC-L or eMDC II) is a proteolytically active ADAM that is highly expressed in the epididymis and in lymphocytes [65-67]. Several alternatively spliced forms of ADAM28 have been detected *in vivo*, including a soluble form without a transmembrane region or cytoplasimc tail [66,67]. ADAM7, although proteolytically inactive, is closely related to ADAM28 (Figure 1). Genes encoding ADAM7, ADAM28, and ADAMDEC1 (Decysin) form a metalloproteinase gene cluster on human chromosome 8p12, presumably as a result of gene duplication [68]. ADAMDEC1 is a soluble ADAM-like protein lacking part of the disintegrin domain and the entire cycteine-rich domain; a conserved histidine residue in the zinc-binding motif is replaced by aspartate, but such a replacement was thought to have no negative effect on the metalloproteinase activity [68]. Expression of ADAMDEC1 is restricted to the immune system and is regulated by various stimuli during monocyte differentiation [69].

As discussed above, no *Xenopus *orthologue of ADAM7 was identified in this analysis. ADAMDEC1 seems to exist only in mammals [12], and we were unable to identify any likely orthologue in the *X. tropicalis *genome or in *X. tropicalis*/*X. laevis *EST databases. However, a BLAST search against the *X. tropicalis *genome assembly yielded four potential genes possibly encoding ADAM28 homologues on Scaffold_30 (Figure 2K). Although these potential genes have only slightly higher sequence similarities to ADAM28 than to ADAM7, the deduced protein sequences all contain a conserved zinc-binding motif (Additional File 3 and data not shown). Therefore they are considered homologues of *adam28 *(as ADAM7 is proteolytically inactive and seems to be restricted to mammals), and were assigned the names *adams 28a-d*. We obtained full-length cDNA clones for *adams 28a *and *28b*, and found two partial clones for *adam28c *(CX934006 and CF377167). In addition, we identified a full-length cDNA encoding a soluble form of ADAM28b (ADAM28bs) in *X. laevis *EST databases (Additional File 3). The presence of soluble ADAM28 in both humans and frogs indicates a potentially conserved function of a diffusible ADAM28 metalloproteinase *in vivo*. A sequence alignment for frog and mammalian ADAM28 (including the soluble forms) is shown in Additional File 3. As in the case of human chromosome 8, the cluster of *adam28 *homologue genes on Scaffold_30 of the *X. tropicalis *genome may also represent gene duplication events in this region. Sequence comparison of the four *X. tropicalis *ADAM28 homologues with human ADAM7 and ADAMDEC1 nevertheless indicates no orthologous relationship between them (not shown). Therefore these duplication events in the frog and human genomes may have happened separately after the divergence of amphibians from mammals. Consistent with this hypothesis, both chicken and fish genomes lack such duplication in this region (Figure 2K).

### ADAM9

ADAM9 (MDC9) was among the three active ADAM metalloproteinases (the other two were ADAMs 12 and 19) first identified in myoblasts. Because one of these ADAMs, ADAM12, is required for myotube formation in a myoblast cell line and in certain muscle tissues *in vivo*, these three ADAMs were also called the meltrins [14,70]. However, ADAM9 is not the most closely related paralogue of ADAMs 12 and 19 (Figure 1), and no apparent phenotype in muscle development was observed in *adam9*^*-/- *^mice, although a recent report showed that they eventually develop retinal degeneration [47]. These findings suggest that ADAM9 does not belong to the meltrin clade.

**Figure 5:**
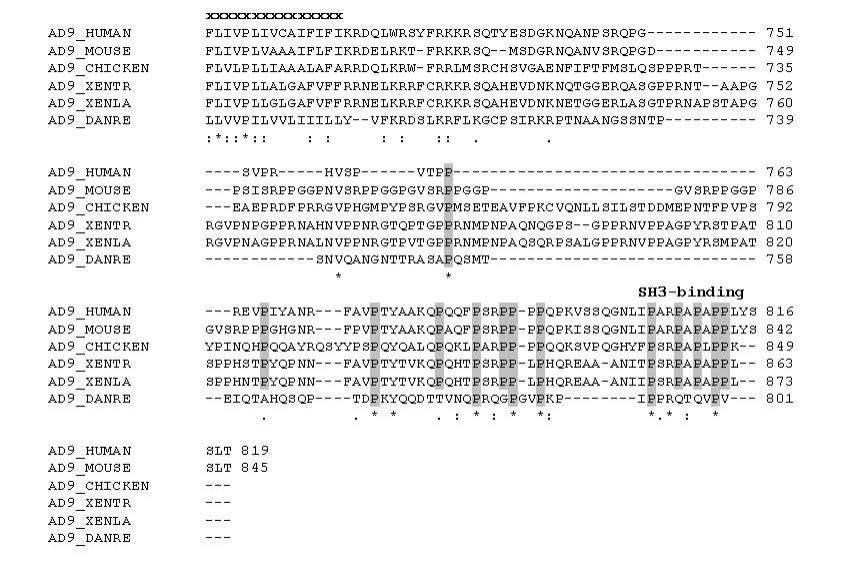
**Sequence comparison of the cytoplasmic tails of ADAM9 from representative vertebrate species**. Sequence alignment of human, mouse, chicken, *X. tropicalis*, *X. laevis *and zebrafish ADAM9 was generated using ClustalX. Residues in the transmembrane region are indicated with "x", and the conserved proline residues (including those in the putative C-terminal SH3-binding motif) are highlighted in grey. Identical, conserved, and semi-conserved residues are indicated with "*", ".", and ":", respectively. See Additional File 4 for complete sequence alignment.

An *X. laevis *orthologue of ADAM9 (xMDC9) was cloned in a previous study and, like its mouse orthologue, was found to be ubiquitously expressed at different developmental stages [43]. In this study we identified a full-length EST clone encoding the putative *X. tropicalis *orthologue of ADAM9. Like their mammalian and zebrafish orthologues, both *X. tropicalis *and *X. laevis *ADAM9 proteins contain a proline-rich cytoplasmic tail (Figure 5 and Additional File 4). Two SH3 binding motifs have been identified previously in the cytoplasmic tails of human and mouse ADAM9, of which the C-terminal proximal one is also conserved in all the tetrapod species examined, including both *X. tropicalis *and *X. laevis *(Figure 5).

### ADAMs 11, 22 and 23

Three proteolytically inactive ADAMs, ADAMs 11, 22 and 23 (also known as MDC, MDC2 and MDC3, respectively), form an ADAM clade that is highly expressed in the brain [71]. Studies with knockout mice revealed that each of these ADAMs is required for normal neuronal function [15,16,49,50]. It was recently shown that ADAM22 functions as a receptor for the secreted protein LGI1. Mutations in LGI1 cause heritable epilepsies in humans, and one of these mutated forms of LGI1 fails to bind ADAM22 [29,72]. In a separate study, ADAMs 11, 22 and 23 were all shown to interact with LGI1 and the closely related LGI4 [28], suggesting that there may be a common mechanism for these ADAMs in maintaining normal brain function. Two *X. laevis *cDNA clones encoding different ADAMs, one partial (for xMDC11a) and one full-length (for xMDC11b), were described previously [43]. Our sequence comparison with the *X. tropicalis *and human genomes indicates that they are orthologues of mammalian ADAMs 11 and 22, respectively (not shown). *X. laevis *ADAM11 is highly expressed in CNC cells [43], but no functional study for this ADAM has been reported to date.

**Figure 6:**
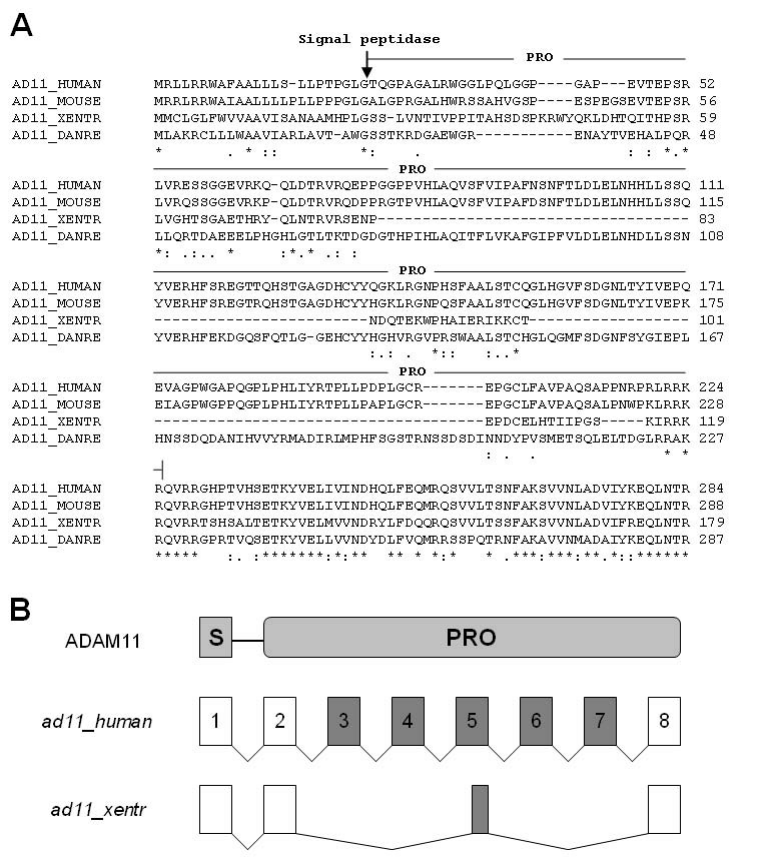
**The truncated propeptide of *X. tropicalis *ADAM11 as a result of different exon usage**. A) Sequence alignment of human, mouse, *X. tropicalis *and zebrafish ADAM11 was generated using ClustalX. Only the N-terminal signal peptide and propeptide are shown; arrow points to predicted signal peptide cleavage sites. See Additional File 5 for complete sequence alignment. B) Comparison of exon-intron structures in the signal (S) and propeptide (PRO) encoding regions of human and *X. tropicalis adam11 *transcript. Open boxes represent exons conserved in both species, and filled boxes represent exons in human *adam11 *that are completely or partially absent in *X. tropicalis adam11*.

Here we report the full-length cDNA sequences for *X. tropicalis *ADAMs 11, 22 and 23. As shown in Table 1, these three *X. tropicalis *ADAMs resemble their human orthologues in both the deduced protein sequence and the high expression level in the brain [71]. ADAM11 is dispensable for survival in mice, but has important roles in spatial learning, motor coordination and nociceptive response [15,16]. Like its mammalian orthologues, *X. tropicalis *ADAM11 also has a very short cytoplasmic tail (Additional File 5). However, it lacks a large portion of the propeptide that is present in human, mouse and zebrafish ADAM11 (Figure 6A and Additional File 5). A survey of the exon-intron boundaries suggests that this difference arises from adoption of different splice sites, as exons 3, 4, 6, 7 and part of exon 5 in human *adam11 *(and mouse and zebrafish *adam11*; data not shown) are absent in the *X. tropicalis adam11 *transcript, while the other exons are conserved between these two species (Figures. 1B and 6B). Because ADAM11 does not have the zinc-binding motif that is required for metalloproteinase activity, it is conceivable that part of the ADAM11 propeptide may have been lost in frogs during evolution.

**Figure 7:**
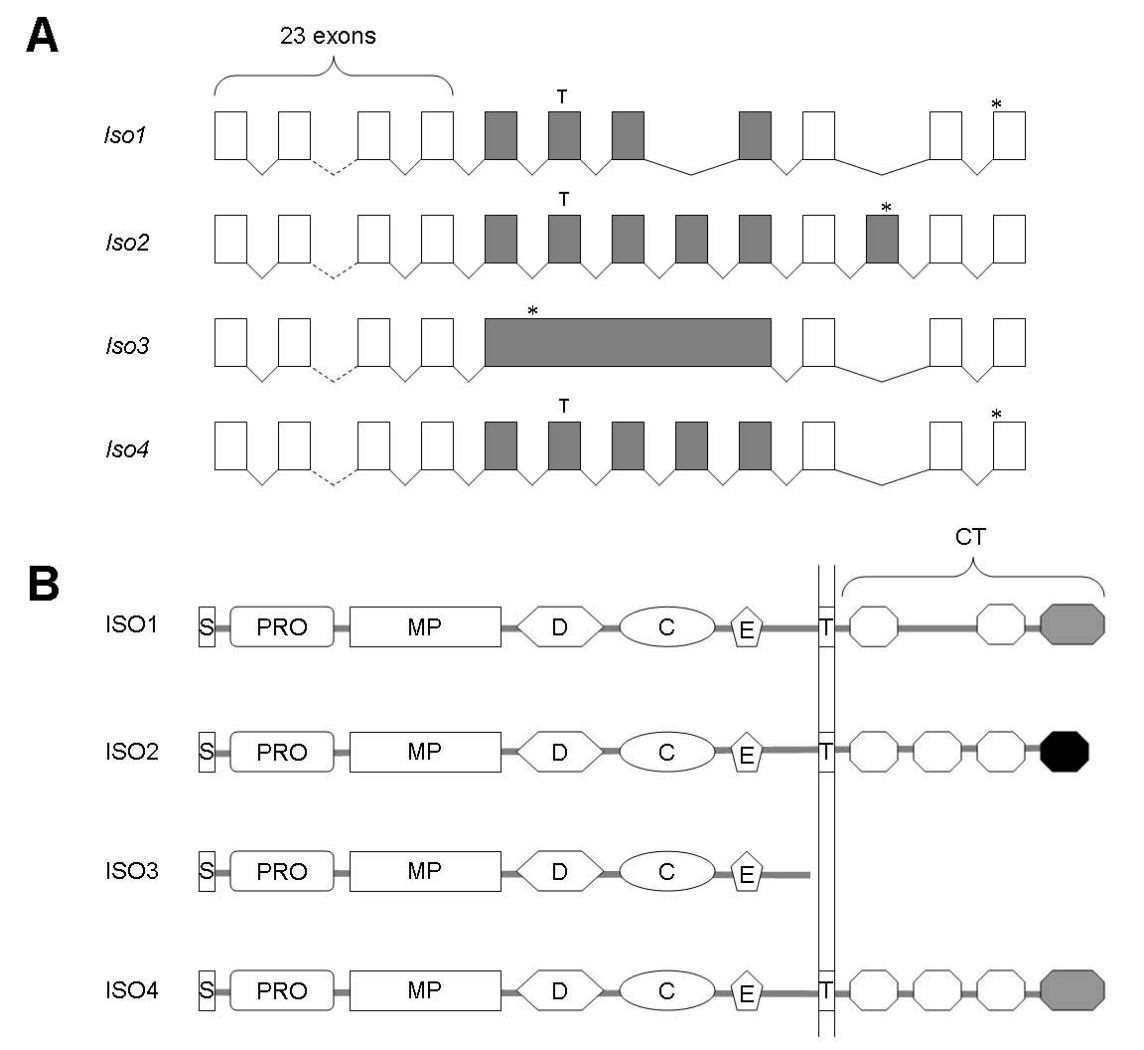
**Alternative splicing of *Xenopus adam22 *transcripts**. A) Exon usage of three *X. tropicalis *isoforms (*Iso1-3*) and one *X. laevis *isoform (*Iso4*) of *adam22 *mRNA. Splicing of the *X. laevis *isoform was predicted by alignment with *X. tropicalis *genome sequence. Open boxes represent exons shared by all four isoforms, and filled boxes represent alternatively spliced exons. Stop codons and nucleotides encoding the transmembrane region are indicated with "*" and "T", respectively. B) Domain organization of the four *Xenopus *isoforms of ADAM22 protein. The C-terminal region shared by ISOs 1 and 4 is shown in grey, and the unique C-terminal region of ISO2 is shown in black. See Figure 1 legend for abbreviations of domain names.

Mice lacking either ADAM22 or 23 develop severe ataxia and die shortly after birth, and the similarity in phenotype has led to the speculation that these two ADAMs may act together in a nonredundant way [49,50]. Because of the similar abilities of ADAMs 11, 22 and 23 to interact with LGIs, it was also proposed that distinct functions of these ADAMs may depend on their expression in different cell types [28]. Interestingly, both human and mouse *adam22 *transcripts have multiple alternatively spliced forms, and mouse cDNA libraries obtained from different types of cells contain different splice variants of *adam22 *[49,73]. We identified three differently spliced isoforms of ADAM22 in *X. tropicalis*, and sequence comparisons revealed that the previously published *X. laevis *ADAM22 (xMDC11b; O42596) represents yet another isoform (Figure 7A). In mammals, alternative splicing results in multiple transmembrane ADAM22 variants with different cytoplasmic tails; however, no soluble form has been reported to date [49,73]. Among the four *Xenopus *ADAM22 isoforms identified in this study, three (ISOs 1, 2 and 4) are transmembrane forms that differ in their cytoplasmic tails. Interestingly, the other isoform (ISO3) is a truncated variant that lacks a transmembrane region and a cytoplasmic tail (Figure 7B). Given the potential physiological role of ADAM22 as a cell surface receptor, this soluble form may provide a possible regulatory mechanism to control the functions of the transmembrane forms of ADAM22. It will be of interest to determine if the splicing of *adam22 *mRNA in *Xenopus *is also cell-type specific.

### ADAMs 10, 10-like and 17

ADAMs 10 and 17 are probably the most extensively studied ADAMs because of their important roles in development and disease. ADAM10 (also known as Kuzbanian or KUZ) was originally discovered in *Drosophila *as an essential component of the Notch signaling pathway [74], while ADAM17 (also known as tumor necrosis factor-α converting enzyme or TACE) was first cloned in mice as the major enzyme required for shedding of the cytokine TNF-α [75,76]. ADAMs 10 and 17 share many substrates, and it is still a subject of debate as to which of these two enzymes is required for cleaving Notch receptors [77,78]. The *Xenopus *system has long been used to study Notch signaling, and ectopic expression of a dominant-negative form of mouse ADAM10 was shown to cause a Notch phenotype in *X. laevis *embryos [26]. Therefore it is important to identify the *Xenopus *homologues of ADAMs 10 and 17 and understand their respective roles in early development.

**Figure 8:**
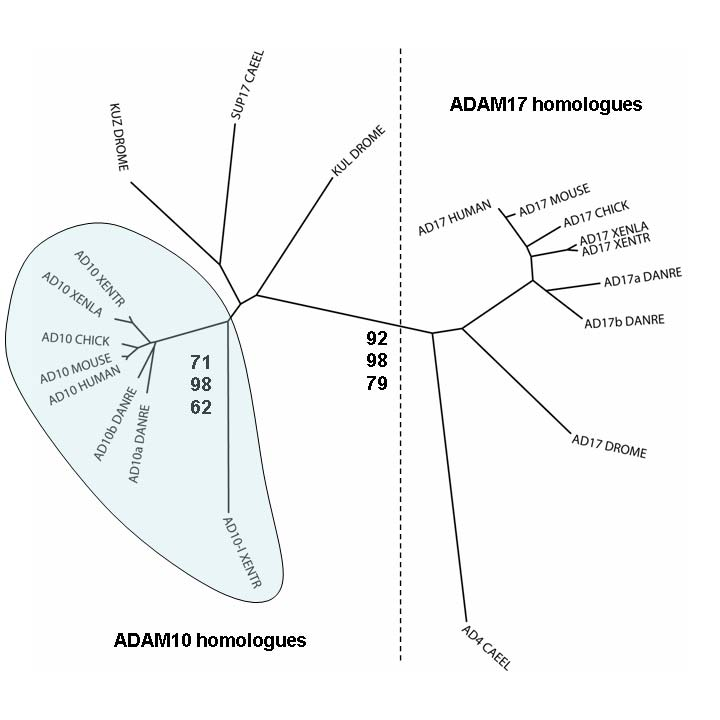
**Phylogenetic analysis of ADAM10 and 17 homologues in vertebrates and invertebrates**. ADAM10 and 17 homologues from *C. elegans *(CAEEL), *Drosophila *(DROME), chicken, zebrafish, frogs (XENTR and XENLA), mouse and human were aligned, and a neighbor-joining tree was drawn using Clustal X. The vertebrate ADAM10/ADAM10-like clade is highlighted, and numbers are bootstrap values (in percentage) from Neighbor (top), Protpars (middle), and Proml (bottom) analyses (see Additional File 6, G-I for trees generated using these models). AD10-l, ADAM10-like.

We identified cDNA clones for both *X. tropicalis *and *X. laevis *ADAM17 from *Xenopus *EST collections using BLAST searches. Although it is not clear if the *X. tropicalis *genome contains a true TNF-α gene, these *Xenopus *ADAM17 proteases may still be involved in cleavage of other members of the TNF superfamily and/or ligands of the epidermal growth factor receptor, as their mammalian orthologues do [79]. In addition to our previous identification of *X. laevis *ADAM10 [7], we obtained a full-length clone encoding *X. tropicalis *ADAM10. A pair-wise sequence comparison shows 82% identity between *X. tropicalis *and human ADAM10, the highest among all ADAMs identified in this study (Table 1), indicating that functions of this enzyme are likely conserved in frogs. In the *Drosophila *genome there exist two ADAM10 homologues, Kuzbanian (KUZ) and Kuz-like (KUL), both of which are required for Notch signaling in certain cells and tissues [74,80]. We also found an actively transcribed paralogue of ADAM10 in *X. tropicalis*, which we named ADAM10-like (Table 1). However, sequence comparisons suggest that ADAM10-like is not the orthologue of *Drosophila *KUL (Figure 8). Therefore these two ADAM10 paralogues in different species may have been derived by two independent gene duplication events. Homologues of ADAM10-like were found in some other animals, including *X. laevis*, zebrafish and a few mammalian species. Detailed sequence analysis and functional studies of ADAMs 10, 10-like and 17 will be reported elsewhere (Xu et al., manuscript in preparation). A phylogenetic tree estimating the evolution of ADAMs 10 and 17 (as well as KUL and *X. tropicalis *ADAM10-like) is shown in Figure 8.

## Conclusions

ADAMs are widely involved in developmental and pathological processes through their activities as proteases and binding partners for other proteins. The recent emergence of *Xenopus tropicalis *as a powerful new vertebrate model for developmental and genetic studies prompted us to take a genome-wide approach to identify ADAM genes existing in this species. Using bioinformatics tools, we were able to obtain full-length sequence information for putative *X. tropicalis *orthologues of most mammalian non-testis specific ADAMs. The orthology between *X. tropicalis *and human ADAMs were further supported by conserved splicing patterns and synteny. The high sequence similarities between mammalian and *Xenopus *ADAMs 9, 10, 11, 12, 17, 19, 22 and 23 are in good agreement with their essential roles *in vivo*, as demonstrated by previous studies with knockout mice. Our sequence analyses also revealed the conservation of certain features, such as alternative splicing and cytoplasmic SH3 binding motifs, in some ADAMs. Functional characterization of these ADAMs (and specific motifs) in *X. tropicalis *may provide useful information to guide future research on human development and diseases. Evolutionary divergence is also evident by the potential loss of some ADAM genes and ADAM domains/motifs in *Xenopus*. Orthologues of ADAMs 7 and 8 were not detected in the current version of *X. tropicalis *genome sequence, and ADAM15 protease activity and part of the ADAM11 propeptide seem to be absent in *X. tropicalis*. These findings suggest that functions of these ADAMs or ADAM domains are likely not conserved. In contrast, the additional ADAM10- and ADAM28-like metalloproteinases, which presumably arose as results of gene duplication, may have conferred selective advantages during the evolution of amphibians. The sequence information described in this study provides the basis for understanding the evolution of ADAM functions in vertebrates.

## Methods

### Bioinformatics

Homologues of known human or *X. laevis *ADAM genes were identified by using sequence-based searches of the Joint Genome Institute (JGI) *X. tropicalis *genome assembly v4.1 [81], and cDNA constructs were obtained from IMAGE Consortium distributor (OpenBiosystems) or by experimental cloning as described below. All new *X. tropicalis *ADAM cDNAs reported here have been sequenced by the Biomolecular Research Facility at the University of Virginia, and assembled to give the final full-length sequences. Multiple sequence alignments were constructed with ClustalX 2.0 [82]. Phylogenetic trees were generated using ClustalX (neighbor joining), Phylip neighbor, Phylip protpars (maximum parsimony) and Phylip proml (maximum likelyhood) [83]. Trees were displayed using the Phylip application Drawtree. Because phylogenetic trees generated using different methods are consistent with each other, only those generated with ClustalX are shown in the main figures (see Additional File 6 for trees generated using the other models). For bootstrap analyses, bootstrap replicate groups were created using Phylip application Seqboot, and bootstrap values were calculated using Phylip Neighbor, Protpars, and Proml analyses (bootstrap values calculated with ClustalX are very similar to those calculated with Phylip Neighbor, therefore only the latter are shown). Synteny analyses were carried out using Metazome [84]. Exon-intron structures were estimated by comparing the cDNA sequences with *X. tropicalis *genome sequence using BLAT searching from the UCSC Genome Bioinformatics Site [85]. Signal peptidase cleavage sites were assigned according to available sequence annotations (for known ADAMs) or predictions by SignalP (for new ADAMs identified in this study) [86]. Domain structure predictions were carried out using the Simple Modular Architecture Research Tool (SMART) [87].

The following human ADAM protein sequences were used to identify their *X. tropicalis *homologues: ADAM9 (Q13443), ADAM10 (O14672), ADAM11 (O75078), ADAM12 (O43184), ADAM15 (Q13444), ADAM17 (P78536), ADAM19 (Q9H013), ADAM22 (Q9P0K1), ADAM23 (O75077), ADAM28 (Q9UKQ2), and ADAM33 (Q9BZ11). Previously reported *X. laevis *ADAMs used in this study are: ADAM9/xMDC9 (NP_001079073), ADAM10 (Q8JIY1), ADAM11/xMDC11a (Q9PSZ3), ADAM13 (AAI69959), ADAM19 (ACE82293), and ADAM22/xMDC11b (O42596). IMAGE clone IDs for ESTs encoding *X. tropicalis *and *X. laevis *ADAMs identified in this study are: *X. tropicalis *ADAM9 (7644280), ADAM10 (7644709), ADAM11 (7650432), ADAM15 (7693815), ADAM17 (6994025), ADAM22 isoform 1 (7658598), ADAM22 isoform 2 (8916700), ADAM22 isoform 3 (7650959), ADAM23 (7649052), ADAM28a (7716115), and ADAM28b (8898681); *X. laevis *ADAM15 (7978429; GenBank accession # BC146626), ADAM17 (3199617; GenBank accession # DQ287907), and ADAM28bs (5513111; GenBank accession #BC091726).

### Cloning and RT-PCR

Total RNA from *X. tropicalis *embryos at desired stages was prepared as described [88]. *X. tropicalis *orthologues of *X. laevis adam13 *and mammalian *adams 12 *and *19*, and *X. tropicalis adam10-like *gene were identified by searching against the *X. tropicalis *genome assembly, and the predicted sequences were used to design primers for RT-PCR to obtain partial clones. 5'- and 3'- RACE was then performed using BD Smart RACE kit (BD Biosciences), and full-length cDNA sequences were assembled into pCR2.1. RT-PCR experiments for expression of *adams 12*, *13*, and *19 *were carried out using total RNA purified from *X. tropicalis *embryos at different developmental stages. Primer sequences are shown in Additional File 7.

## Authors' contributions

SW conceived of the study, carried out most of the experiments and data analyses, and drafted the manuscript. CAW performed the sequence alignment and phylogenetic analyses, and helped draft the manuscript. GX, LCB and AS participated in the cloning and RT-PCR experiments. DWD and JMW participated in the design and coordination of this study, and helped draft the manuscript. All authors read and approved the final manuscript.

## Supplementary Material

Additional file 1 **Phylogenetic tree of ADAMs from representative vertebrate species**. Click here for file

Additional file 2 **Complete sequence alignment of ADAM15 from representative vertebrate species**. Click here for file

Additional file 3 **Complete Sequence alignment of ADAM28 from representative vertebrate species**. Click here for file

Additional file 4 **Complete sequence alignment of ADAM9 from representative vertebrate species**. Click here for file

Additional file 5 **Complete sequence alignment of ADAM11 from representative vertebrate species**. Click here for file

Additional file 6 **Phylogenetic trees generated using alternative models**. Click here for file

Additional file 7 **Sequences of primers used in RT-PCR experiments shown in Figure **3B. Click here for file
