# Supplementary material for: Conservation and divergence of ADAM family proteins in the Xenopus genome
Source: BMC Evol Biol. 2010 Jul 14;10:211. doi: 10.1186/1471-2148-10-211 (PMC3055250; doi:10.1186/1471-2148-10-211)
Supplement: Additional file 1 — Phylogenetic tree of ADAMs from representative vertebrate species. [file 1471-2148-10-211-S1.PDF]

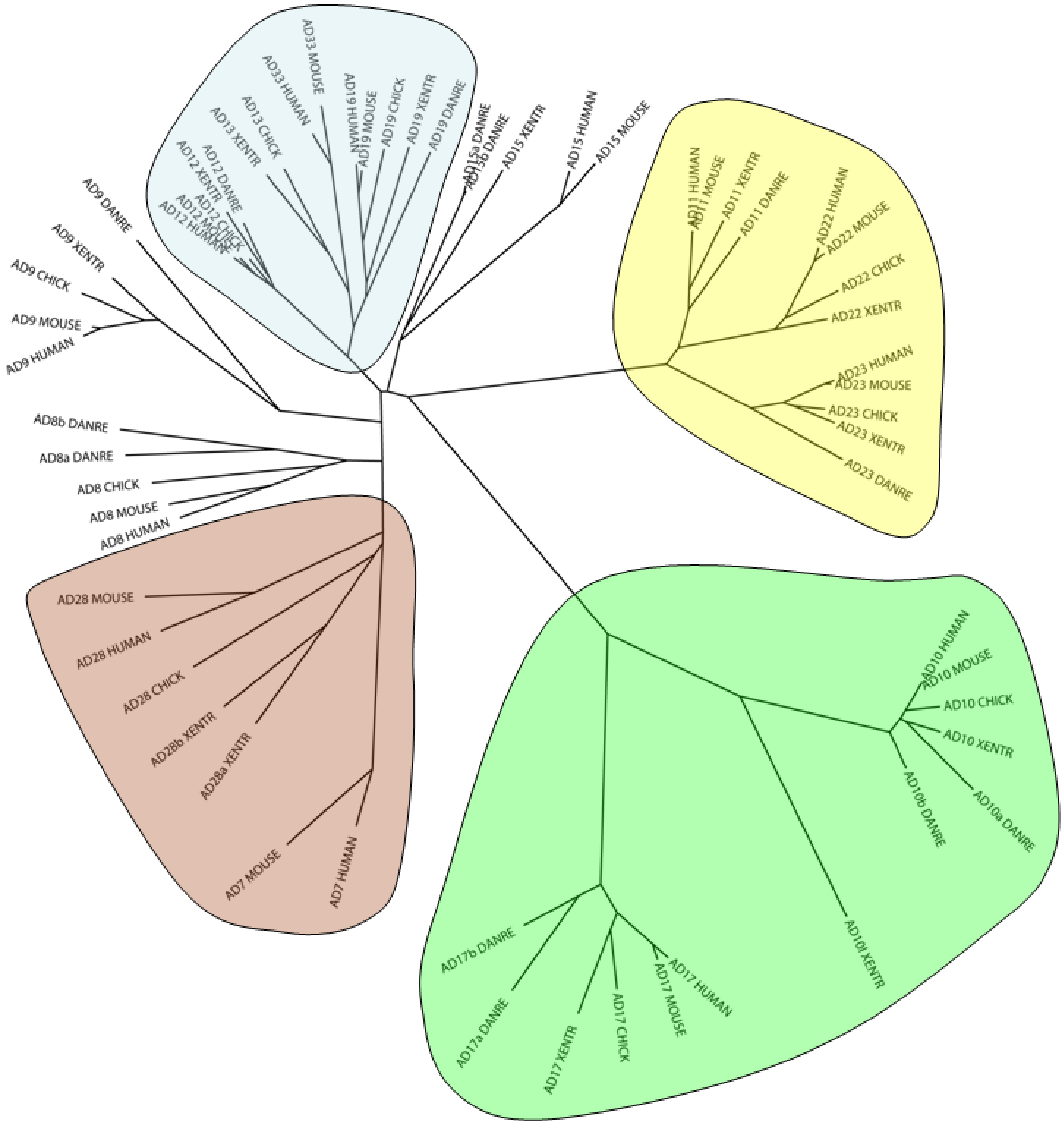

Additional File 1. Phylogenetic tree of ADAMs from representative vertebrate species. Protein sequences of conserved ADAMs from human, mouse, chicken, zebrafish (DANRE) and *X. tropicalis* (XENTR) were aligned, and a neighbor-joining tree was drawn using ClustalX. Clades are highlighted by different colors as in Fig. 1A.
