# Supplementary material for: Conservation and divergence of ADAM family proteins in the Xenopus genome
Source: BMC Evol Biol. 2010 Jul 14;10:211. doi: 10.1186/1471-2148-10-211 (PMC3055250; doi:10.1186/1471-2148-10-211)
Supplement: Additional file 2 — Complete sequence alignment of ADAM15 from representative vertebrate species. [file 1471-2148-10-211-S2.PDF]

```
                          Signal peptidase

AD15_HUMAN       MRLALLWALGLLGAGSPLPSWPLPNIG-----------------GTEEQQAESEKAPREP 43
AD15_PANTR       MRLALLWALGLLGAGSPLPSWPLPNIALLSIPSVLSWGVLGPAGGTEEQQAESEKAPREP 60
AD15_CANFA       MRLALLWALGLLGAGSPLTSRPLADIG-----------------GTEDEHARPERALGGP 43
AD15_BOVIN       MRLALLWALGLLGAGSPLPSRPLPDIG-----------------VTEEMQARPERAQNGP 43
AD15_MOUSE       MRLALLWALGLLGAGSPRPSPPLPNIG----------------GTEEEQQASPERTLSGS 44
AD15_RAT         MRLALLWALGLLGAGSPRPSPPLPNIG----------------GTEEEQQASPERTQSRS 44
AD15_XENLA       MRLGLFRLLQLLLSGLGICGL---------------------------DRVQPDGILSWE 33
AD15_XENTR       MRVGPFRLLQLLLCGLCLG------------------LSTRDRAVSDRVAVQPDSPLYWE 42
AD15a_DANRE      -------------------------------------------------------NLSLS 5
AD15b_DANRE      ------------------------------------------------------------


AD15_HUMAN       LEPQVLQDDLPISLKKVLQTSLPEPLRIKLELDGDSHILELLQNRELVPGRPTLVWYQPD 103
AD15_PANTR       LEPQVLQDDLPISLKKVLQTSLPEPLRIKLELDGDSHILELLQNRELVPGRPTLVWYQPD 120
AD15_CANFA       SEPQILRDHPMLSLAEMLQTNLPEAFRIKLELDGDSHILELLQNRDQVPGRPWLMRYQPD 103
AD15_BOVIN       LEPQILQDNPTLSLAEALQTSLPEALRIKLELDGESHILELLQNRELVSGRPTLVWYQPD 103
AD15_MOUSE       MESRVVQDSPPMSLADVLQTGLPEALRISLELDSESHVLELLQNRDLIPGRPTLVWYQPD 104
AD15_RAT         LENQVVQDSPPINLTEVLQTGLPETLRIGLELDGENHILELQQNRDLVPGRPTLVWYQPD 104
AD15_XENLA       VTPELHRRGHRLSLQDVMRT-LPSDLHMSMEIDGNQVELDLEWNRHLLPESHSLTYYLPN 92
AD15_XENTR       VTPRLHRSGHRLGLQEAMQI-LPDDLQISMEIDGEQVQLDLERNRHLVPGSHRLIYYLPN 101
AD15a_DANRE      LFLSLQKG-------------HPDRLQCGLQVGSSLYILDLEKNQDLLPKPPNVFYYLPN 52
AD15b_DANRE      ---------------------HPDRLQCGLQVGSSLYILDLEKNQDLLPKPPNVFYYLPN 39
                                       *. ::  :::...   *:*  *:. :.    :  * *:

AD15_HUMAN       GTRVVSEGHTLENCCYQGRVRGYAGSWVSICTCSGLRGLVVLTPERSYTLEQGPGDLQGP 163
AD15_PANTR       GTRVVSEGHTLENCCYQGRVRGYAGSWVSVCTCSGLRGLVVLTPERSYTLEQGPGDLQGP 180
AD15_CANFA       GIRVVSEGHTLENCCYQGRVQGHASSWVSVCTCSGLRGLVILSPERSYMLDLGPGDLQAP 163
AD15_BOVIN       GTRVVGEGHTLENCCYEGRVRGHAHSWVSLCTCSGLRGLVILSPERSYSVELGPRDLQGP 163
AD15_MOUSE       GTRMVSEGYSLENCCYRGRVQGHPSSWVSLCACSGIRGLIVLSPERGYTLELGPGDLQRP 164
AD15_RAT         GTRMVSEGHSLENCCYRGRVQGRPSSWVSLCACSGIRGLVVLSPERSYTLELGPGDLQRP 164
AD15_XENLA       GSHVTQRDPRVENCYYHGQLEGQTGPWSSISLCSGIRGIILTSPERGYSIEPLPGDPPHK 152
AD15_XENTR       GSRVTDQDPGVANCYYQGQLEGQTGPWSSLSLCSGIRGIIMTSPERGFSIEPLPGDPSHK 161
AD15a_DANRE      GTGVSMKENTVTHCYYHGSVQGFPESRVALSSCSGLRGVIVINATLSFELLPEGEEERGK 112
AD15b_DANRE      GTGVSMKENTVTHCYYHGSVQGFPESRVALSSCSGLRGVIVINATLSFELLPEGEEERGK 99
                 *  :  .   : :* *.* :.* . .  ::. ***:**::: ..  .: :     :

AD15_HUMAN       PIISRIQDLHLP--------GHTCALSWRESVHTQTPPEHPLGQRHIR--RRRDVVTETK 213
AD15_PANTR       PIISRIQDLHLP--------GHTCALSWRESVHTQTPPEHPLGQRHIR--RRRDVVTETK 230
AD15_CANFA       PNISRIQDLFLP--------GHTCALSRHASGPTQAPLEQPPGQHHSCTRRRRDVVAETK 215
AD15_BOVIN       PVVSRIQDLLLP--------RHTCALSWPTSVLPQALPEPSLGQRHTH-RWRRDVVTETK 214
AD15_MOUSE       -VISRIQDHLLL--------GHTCAPSWHASVPTRAGPDLLLEQHHAH-RLKRDVVTETK 214
AD15_RAT         LIVSRIQDLLLP--------GHTCAPSWHAFVPTEAAPDLLLEQHHLR-RLKRDVVTETK 215
AD15_XENLA       HIVR---LTPG------NSRNSTCGSTEAPQSLK-------LSPHHRI---KRDVVTEAK 193
AD15_XENTR       HIVR---LTQE------NPKNSSCGNSETPHLLTGTIGTQSREPHHRT---KRDVVTERK 209
AD15a_DANRE      DDEGSGEMEEGEMHAIYSTHSPTSESRDCGVSHTSIPPIQIIPHTHRS---KRDILSEIK 169
AD15b_DANRE      DDEGSGEMEEGEMHAIYSTHSPTSESRDCGVSHTSIPPIQIIPHTHRS---KRDILSEIK 156
                                       :.                     *     :**:::* *

AD15_HUMAN       TVELVIVADHSEAQKYRDF-----QHLLNRTLEVALLLDTFFRPLNVRVALVGLEAWTQR 268
AD15_PANTR       TVELVIVADHSEAQKYQDF-----QHLLNRTLEVALLLDTFFQPLNVRVALVGLEAWTQR 285
AD15_CANFA       IIELVIVADHSETQRYPDS-----QQLLNRMLKVTFLLDTFFRPLNVRVVLVGLEAWTQH 270
AD15_BOVIN       IVELVIVADHAEVKRHRDF-----QSLLNRTLEVALLLDTFFRPMNVRVALVGLEAWTQH 269
AD15_MOUSE       IVELVIVADNSEVRKYPDF-----QQLLNRTLEAALLLDTFFQPLNVRVALVGLEAWTQH 269
AD15_RAT         IVELVIVADNSEVRKYPDF-----QQLLNRTLEVALLLDTFFQPLNVRVALVGLEAWTQR 270
AD15_XENLA       YVELVLVADNTEYKMFSGD----MKSLHLRILEIANKVDAFYRPLNVRVALVSVEVWNQK 249
AD15_XENTR       FVELAMVADNREYKLLHGD----MKGLHIRMLEIANKVDAFYRPLNVRVALVAVEVWNQK 265
AD15a_DANRE      YIELVLVADHKESSSQYLNYQKNNKTIIYRMLDVANQVDWFYRPLNVRVALLGLEIWSDQ 229
AD15b_DANRE      YIELVLVADHKEVNKHHLN----NKTIIYRMLDVANQVDWFYRPLNVRVALLGLEIWSDQ 212
                  :**.:***: *            : :  * *. :  :* *::*:****.*:.:* *.::

AD15_HUMAN       DLVEISPNPAVTLENFLHWRRAHLLPRLPHDSAQLVTGTSFSGPTVGMAIQNSICSPDFS 328
AD15_PANTR       DLVEISPNPAVTLENFLHWRRAHLLPRLPHDSAQLVTGTSFSGPTVGMAIQNSICSPDFS 345
AD15_CANFA       DLVEISQDPGLTLDNFLHWRRVDLLPRLPHDSAQLVTATSFSGPTVGMAVQNSICSPDFS 330
AD15_BOVIN       NLIEISQNPGLTLDSFLHWRRTNLLPRLPHDSAQLVTATSFSGPMVGMAIQNSICSPEFS 329
AD15_MOUSE       NLIEMSSNPAVLLDNFLRWRRTDLLPRLPHDSAQLVTVTSFSGPMVGMAIQNSICSPDFS 329
AD15_RAT         DLIEMSSNPAVLLDNFLRWRRTDLLPRLPHDSAQLVTVTSFSGPMVGMAIQNSICSPDFS 330
AD15_XENLA       DQINVTTNPAETLYHFLSWREKNLLPRVTNDNAQLLTGITFMNSSVGMATMNSMCSAGYS 309
AD15_XENTR       DQINISTNPAETLYRFLSWRERSLLPLVANDNAQLLTGLTFKNSSVGMATLNSMCSSGGS 325
AD15a_DANRE      DKIKVDKSPTETLNRFLDWRTRELLPRLRHDNAQLIMGESFDGTTVGMASQSSMCSKDRS 289
AD15b_DANRE      DKIKVDKSPTETLNRFLDWRTRELLPRLRHDNAQLIMGESFDGTTVGMASQSSMCSKDRS 272
                 : :::  .*   *  ** **   *** : :*.***:   :* .. ****  .*:**   *
```

```
                                         Δ
AD15_HUMAN      GGVNMDHSTSILGVASSIAHELGHSLGLDHDLPGNSCPCPGPAPAKTCIMEASTDFLPGL 388
AD15_PANTR      GGVNMDHSTSILGVASSIAHELGHSLGLDHDLPGNSCPCPGPAPAKTCIMEASTDFLPGL 405
AD15_CANFA      GGVNMDHSTSVLGVASSIAHELGHSLGLDHDLPGNSCPCPGPAPAKSCIMEASTDFLPGL 390
AD15_BOVIN      GGVNMDHSTSILGVASSIAHELGHSLGLGHDSPGNSCPCPGPAPAKSCIMQASTDFLPGL 389
AD15_MOUSE      GGVNMDHSTSILGVASSIAHELGHSLGLDHDSPGHSCPCPGPAPAKSCIMEASTDFLPGL 389
AD15_RAT        GGVNMDHSTSILGVASSIAHELGHSLGLDHDSPGNSCPCPGPAPAKSCIMEASTDFLPGL 390
AD15_XENLA      GGVSMDHSVTILGVASTLAHQLGHNLGLSHDT-DRKCGQPSKGKK--WIMEPSGGFLPGL 366
AD15_XENTR      GGVSMDHSVSILGVASTLAHQLGHNLGLSHDT-DRKCGQPSKGKK--WIMEPSAGFLPGL 382
AD15a_DANRE     GGVNVDHLVSVLGVASTVAHELGHNLGMSHDTADRRCHCQNEPRLGGCIMEPSTGFMPGQ 349
AD15b_DANRE     GGVNVDHLVSVLGVASTVAHELGHNLGMSHDTADRRCHCQNEPRLGGCIMEPSTGFMPGQ 332
                ***.:** .::*****::**:***.**:.**  .. *   .       **:.* .*:**

AD15_HUMAN      NFSNCSRRALEKALLDGMGSCLFERLPSLPPMAA-FCGNMFVEPGEQCDCGFLDDCVDPC 447
AD15_PANTR      NFSNCSRRALEKALLDGMGSCLFERLPSLPTMAA-FCGNMFVEPGEQCDCGFLDDCIDPC 464
AD15_CANFA      NFSNCSRQALEKALLDGMGSCLFERLPSLPSMAT-VCGNMFVEPGEQCDCGFPDDCTDPC 449
AD15_BOVIN      NFSNCSRQALEEALLGGMGSCLFERLSGLPSMAS-VCGNMLVEPGEQCDCGFPDECTDPC 448
AD15_MOUSE      NFSNCSRQALEKALLEGMGSCLFERQPSLAPMSS-LCGNMFVDPGEQCDCGFPDECTDPC 448
AD15_RAT        NFSNCSRWALEKALLDGMGSCLFEWPPSRAPMSS-LCGNMFVDPGEQCDCGFPDECTDPC 449
AD15_XENLA      EFSNCSFTDLEFSLRRGGGMCLFNVPPPKRLFGEPQCGNFLVEEGEQCDCGLSQECTDQC 426
AD15_XENTR      EFSNCSLADLEFSLRRGGGMCLFNVPPPKRLFGEPQCGNFLVEEGEQCDCGLTQDCRDPC 442
AD15a_DANRE     LFSSCSERDLSLSLLHGGGMCLFNVPQPESLLGGPRCGNLYVEKGEECDCGLLDECNDPC 409
AD15b_DANRE     LFSSCSERDLSLSLLHGGGMCLFNVPQPESLLGGPRCGNLYVEKGEECDCGLLDECNDPC 392
                 **.**   *. :*  * * ***:       :.   ***: *: **:****: ::* * *
                                                     †
AD15_HUMAN      CDSLTCQLRPGAQCASDGPCCQNCQLRPSGWQCRPTRGDCDLPEFCPGDSSQCPPDVSLG 507
AD15_PANTR      CDSSTCQLRPGAQCASDGPCCQNCQLRPSGWQCRPTRGDCDLPEFCPGDSSQCPPDVSLG 524
AD15_CANFA      CDYFTCQLRPGAQCASNGLCCQNCQLRPAGWQCRPARGDCDLPEFCPGDSSHCPPDVSLG 509
AD15_BOVIN      CDYFTCQLRPGAQCASDGLCCHNCQLRPAGWKCRPTRGDCDLPEFCPGDSSQCPPDVSMG 508
AD15_MOUSE      CDHFTCQLRPGAQCASDGPCCQNCKLHPAGWLCRPPTDDCDLPEFCPGDSSQCPSDIRLG 508
AD15_RAT        CDYFTCQLRPGAQCASDGPCCQNCKLQPAGWQCRLPTDDCDLPEFCLGDSSQCPPDIRLG 509
AD15_XENLA      CESTLCQFRGGAECSSGDQCCEGCKLKVSGSMCREPLGVCDLPEYCNGESPHCPPNVYLQ 486
AD15_XENTR      CDASLCQLRSGAQCSSGDQCCEGCKLKVSGWMCREPLGACDLPEYCNGESPHCPPNVYLQ 502
AD15a_DANRE     CNASTCKLVPGAQCSSDGICCENCKLRVAGSVCREPLGECDLPEHCTGSSPYCPPNVFLQ 469
AD15b_DANRE     CNASTCKLVPGAQCSSDGICCENCKLRVAGSVCREPLGECDLPEHCTGSSPYCPPNVFLQ 452
                *:   *::  **:*:*.. **..*:*: :*  ** . . *****.* *.*. **.:: :
                                                              ‡
AD15_HUMAN      DGEPCAGGQAVCMHGRCASYAQQCQSLWGPGAQPAAPLCLQTANTRGNAFGSCGRNPSGS 567
AD15_PANTR      DGEPCAGGQAVCMHGRCASYAQQCQSLWGPGAQPAAPLCLQTANTRGNAFGSCGRNPSGS 584
AD15_CANFA      DGEPCAGGQAVCVQGRCASYAQQCQALWGPGAQPTAPLCLIAANTRGDAFGSCGRSPNGS 569
AD15_BOVIN      DGEPCASGQAVCMQGRCASYAQQCQALWGPGAKPAAPLCLLTANTRGDAFGSCGRNPDGS 568
AD15_MOUSE      DGEPCASGEAVCMHGRCASYARQCQSLWGPGAQPAAPLCLQTANTRGNAFGSCGRSPGGS 568
AD15_RAT        DGEPCASGEAVCMHGRCASYTRQCQSLWGPGAQPAAPLCLQTANTRGNAFGSCGRSPSGS 569
AD15_XENLA      NGETCDQG--YCYQGECRTIQAQCKDLWGPGSSPAPDPCFSKVNIRGDKYGNCGRSLNGT 544
AD15_XENTR      NGETCDQG--YCYRGECRTLRAQCLDVWGPGSAPAPDPCFSKVNVRGDKYGNCGRSPDGT 560
AD15a_DANRE     NGEPCKGGSSYCYSGVCASLDDQCQMLWGQNSTRAPPICFSSVNKQGNKYGNCGQMPNGS 529
AD15b_DANRE     NGEPCKGGSSYCYSGVCASLDDQCQMLWGQNSTRAPPICFSSVNKQGNKYGNCGQMPNGS 512
                :**.*  *   *  * * :   **  :** .:  :.  *:  .* :*: :*.**:  .*:

AD15_HUMAN      YVSCTPRDAICGQLQCQTGRTQPLLGSIRDLLWETIDVNGT--ELNCSWVHLDLGSDVAQ 625
AD15_PANTR      YVSCTPRDAICGQLQCQTGRTQPLLGSIQDLLWETIDVNGT--ELNCSWVHLDLGSDVAQ 642
AD15_CANFA      YMSCAPKDAMCGQLQCQGGQAQPLLGSARDLHWEVLEANGTQPRLNCSWVHLDLGDDVAQ 629
AD15_BOVIN      YVSCAPRDAMCGQLQCQGGRAQPLLGSARDLRWEMLEANGTQLRLNCSWVHLDLGNDVAQ 628
AD15_MOUSE      YMPCAPRDVMCGQLQCQWGRSQPLLGSVQDRLSEVLEANGT--QLNCSWVDLDLGNDVAQ 626
AD15_RAT        YMPCNLRDAICGQLQCQWGRNQPLLGSVQDQLSEVLEANGT--QLNCSWVDLDLGNDVAQ 627
AD15_XENLA      YLPCAERDVWCGKIQCQGGNSRSRLGPGAQTVLVSVTINGS--ELSCRGTHFDVGDDIWD 602
AD15_XENTR      YLPCAESDVWCGKIQCQGGSARSRLGAGAQTVLVSVTINGS--ELSCRGTHFDLGDDIWD 618
AD15a_DANRE     YIPCLKWDVHCGRIQCQGGGERPLLGSNAEILTTTVILNQS--DFTCRGTYFNLGDDVSD 587
AD15b_DANRE     YIPCLKWDVHCGRIQCQGGGERPLLGSNAEILTTTVILNQS--DFTCRGTYFNLGDDVFD 570
                *:.*   *. **::*** *  :. **.  :     :  * :   :.*  . :::*.*: :

AD15_HUMAN      PLLTLP-GTACGPGLVCIDHRCQRVDLLGAQECRSKCHGHGVCDSNRHCYCEEGWAPPDC 684
AD15_PANTR      PLLTLP-GTACGPGLVCIDHRCQRVDLLGAQECRSKCHGHGVCDSNRHCYCEEGWAPPDC 701
AD15_CANFA      PLLALP-GTACGPDLVCIDHQCQPVDVLRAQECRSKCHGHGVCDSKGHCHCEEGWAPPDC 688
AD15_BOVIN      PLLTLP-GTACGPGLVCVEQQCQPIEVLGAQECQRRCHAHGVCDSNRRCHCEEGWAPPDC 687
AD15_MOUSE      PLLALP-GTACGPGLVCIGHRCQPVDLLGAQECRRKCHGHGVCDSSGHCRCEEGWAPPDC 685
AD15_RAT        PLLALP-GTACGPGLVCIGHRCQPVDLLGAQECRSKCHGHGVCDSSRHCHCDEGWAPPDC 686
AD15_XENLA      SAILVATGTPCGAGKVCVGHKCEDVSKLKVQNCRSKCNGHGVCNSNRNCHCDAGWAPPDC 662
AD15_XENTR      SAVLVATGTPCGTGKVCIGQKCEDVSRLKVQNCRNKCNGRGVCNSNSNCHCDPGWAPPDC 678
AD15a_DANRE     PAMVLQ-GTACGPNKACVDQKCRDVSMFGVDECRSKCHGHGVCNSNKNCHCDEGWAPPDC 646
AD15b_DANRE     PAMVLQ-GTACGPNKACVDQKCRDVSMFGVDECRSKCHGHGVCNSNKNCHCDEGWAPPDC 629
                . : :  **.**.. .*: ::*. :. : .::*: :*:.:***:*. .* *: *******
```

```
                                             xxxxxxxxxxxxxxxxxx
AD15_HUMAN      TTQLKATSS--------------LTTGLLLSLLVLLVLVMLGASYWYRARLHQRLCQLKG 730
AD15_PANTR      TTQLKATSS--------------LTTGLLLSLLVLLVLVMLGASYWYRARLHQRLCQLKG 747
AD15_CANFA      TSHVRATSS--------------LTTGLPLSLLLVVVLVLLGASYWHRARLRQRLCQLKG 734
AD15_BOVIN      TTHVRATSS--------------LTTGLPLSLLLLLVLVLLGASYWHRARLHQRLCQLKG 733
AD15_MOUSE      MTQLKATSS--------------LTTGLLLSLLLLLVLVLLGASYWHRARLHQRLCQLKG 731
AD15_RAT        MTQLRATSS--------------LTTGLLLSLLLLLVLVLLGASYWYRARLHQRLCQLKG 732
AD15_XENLA      AASGQGGSIDSGSVPLKNVGNSRTSALLMIFLLIVPLLILLSICYWKRNSLQLRLGKLSP 722
AD15_XENTR      VGSGQGGSIDSGSLSGRPDGSSVTAALLVLFLLVVPLLVLLGICYIKRDTLQRRLGKFST 738
AD15a_DANRE     RYSGNGLNT-----------IQMRLIKLLVCFFCVPCSLNAYFFFRKLYEVLCGYMWFLM 695
AD15b_DANRE     RYSGNGG----------------------------------------------------- 636
                    ..

AD15_HUMAN      PT-CQYRAAQ----SGPSERPGPPQRALLARGTK-------------------------- 759
AD15_PANTR      PT-CQYRAAQ----SGPPERPGPPQRALLAQGTK-------------------------- 776
AD15_CANFA      PS-CQYRAAQ----SGPSECPGPPQRVLLMPGAKPASCF--------------------- 768
AD15_BOVIN      PS-CQYRAAQ----SGPPERPGPPQRALVMPGAK-------------------------- 762
AD15_MOUSE      SS-CQYRAPQ----SCPPERPGPPQRAQQMTGTKQASVVSFPVPPSRPLPPNPVPKKLQA 786
AD15_RAT        SS-CQYRAAQ----SGPPERPGPPQRAQQMPGTK-------------------------- 761
AD15_XENLA      SSKCQYRGAQTSDNQSRPQRPPPPMRTQSTELQVMSTYN--------------------- 761
AD15_XENTR      SSKCQYRVAQTGNDPSRPQRPPPPNWAQGTELQVMSTHS--------------------- 777
AD15a_DANRE     RSRSGTSGLTRVTSPSPRQSASYRTGQRQILAAHWPAPP--------------------- 734
AD15b_DANRE     ---SVDSGP----AQEPRDSDPARVALLVIFLFVLPVSL--------------------- 668
                   .             :

AD15_HUMAN      -----------------------SQGPAKPPPPRKPLPADPQGRCPSGDLPGPGAGIPPL 796
AD15_PANTR      -----------------------SQGPAKPPPPRKPLPADPQGRCPSGDLPGPGAGIPPL 813
AD15_CANFA      --------------------LSHPQGPAKPPPPRKPLPANPQGRGPSGDLPGPGAGIPPL 808
AD15_BOVIN      -----------------------SQGPTKPPPPRKPLPADPHGRRPSGDLPGPGAGIPPP 799
AD15_MOUSE      ALADRSNPPTRPLPADPVVRRPKSQGPTKPPPPRKPLPANPQGQHPPGDLPGPGDGSLPL 846
AD15_RAT        -----------------------PQGPTKPPPPRKPLPANPQGRPPLGDLPGPGDGSLQL 798
AD15_XENLA      ----------------------KPPVPRKPPPPKKPLPLDPSSQAPL--LSVPAYPDHMI 797
AD15_XENTR      ----------------------KPPVPGKPPPPKKPLPLDPLSQAPL--LTVPAYPDHMI 813
AD15a_DANRE     ----------------------TKPLPPDPAPSESKMQCKPTGARPAA-PSKPLPPDPVL 771
AD15b_DANRE     ----------------------LFVALR-------------------------------- 674

AD15_HUMAN      VVPSRPAPPPPTVSS-LYL--- 814
AD15_PANTR      VVPSRPAPPPPTVSS-LYL--- 831
AD15_CANFA      VVPSRPAPPPPAASSSLYL--- 827
AD15_BOVIN      VVPSRPAPPPPAASS-PYL--- 817
AD15_MOUSE      VVPSRPAPPPPAASS-LYL--- 864
AD15_RAT        VVPSRPAPPPPAASS-LYL--- 816
AD15_XENLA      SAPSRPAPPPPHSQRAQQV--- 816
AD15_XENTR      AAPSRPAPPPPHSDRAQQV--- 832
AD15a_DANRE     SSRQNPVPPKPPVPKKPLPVDP 793
AD15b_DANRE     ----------------------
```

Additional File 2. Complete sequence alignment of ADAM15 from representative vertebrate species. Sequences of human, chimpanzee (PANTR), canine (CANFA), bovine (BOVIN), mouse, rat, *X. laevis* (XENLA), *X. tropicalis* and zebrafish (partial without N-terminus) ADAM15 proteins were aligned using ClustalX. Amino acid residues with different properties are colored differently. Identical, conserved, and semi-conserved residues are indicated with "*", ".", and ":", respectively. The highly conserved signal peptide in mammalian ADAM15 proteins, the zinc-binding motif (indicated with "Δ"; the Gln residues in *Xenopus* ADAM15 are underscored) and methionine-turn in the catalytic center, the two RGD integrin binding sites (indicated with "†" and "‡"), and the conserved cytoplasmic proline residues are highlighted in grey. Arrows point to signal peptide cleavage sites, and residues in the transmembrane region are indicated with "x".
