# Supplementary material for: Conservation and divergence of ADAM family proteins in the Xenopus genome
Source: BMC Evol Biol. 2010 Jul 14;10:211. doi: 10.1186/1471-2148-10-211 (PMC3055250; doi:10.1186/1471-2148-10-211)
Supplement: Additional file 3 — Complete Sequence alignment of ADAM28 from representative vertebrate species. [file 1471-2148-10-211-S3.PDF]

```
                                   Signal peptidase
                                          ▼
AD28_HUMAN        MLQG-LLPVSLLLS-VAVS---AIKELPGVKKYEVVYPIRLHPLHKREAKEPEQQEQFET 55
AD28_MOUSE        MQQWSLLVVSFLLSPVPVS---AIKELPKAKKYEVVYPIRLHPLRKRETQEPEPKETFET 57
AD28_CHICK        -MNNVILIFVVLLCFLHQG--SARKKLLGVEQYEIVYPRKLHTVHKRDAERSKEETKYDD 57
AD28a_XENTR       ----MLLPLLLLTAGLLLPQPAASQKLTIDPYYEVVFPKKIHAQHKRDVQG-----MYPE 51
AD28b_XENTR       -MAQAVVPTTLLLSVLLTGHILAFDRIPPGQKYEVVFPKKLHFQQKRDTQS-----KYPS 54
AD28bs_XENLA      -MLQAVLPTALLLSTLFSGHILAFDRIPPGHKYEVVFPRKLHFQHKRDTQS-----KYPN 54
AD28s_HUMAN       MLQG-LLPVSLLLS-VAVS---AIKELPGVKKYEVVYPIRLHPLHKREAKEPEQQEQFET 55
                       ::    .* . :       *  ..:      **:*:* ::*  :**:.:        :

AD28_HUMAN        ELKYKMTINGKIAVLYLKKNKNLLAPGYTETYYNSTGKEITTSPQIMDDCYYQGHILNEK 115
AD28_MOUSE        ELRYKMTVNGKVAVLYLKKNNKLLAPDYSETYYNSSGNKVTTSPQIMDSCYYQGHIVNEK 117
AD28_CHICK        TLEYGIKANGEEVILHLQKNKHLLAGDYTETVYSDDGQQITTSPQIKDHCYYEGYIQDEA 117
AD28a_XENTR       VVQYGIHMGGKHLILHLEKNEDLLSANYTETYYLSDGTPVTTSPKIQNHCYYQGNVKNDN 111
AD28b_XENTR       TVHYEVQLEGEPLVLKLEKTEDLISEHYTETQYLPDGTPIETSPEIQDHCYYQGGVENDS 114
AD28bs_XENLA      TVQYEVQLEGEPLVLKLEKTEDLISEDYTETQYLPDGTPKETSPEIQDHCYYQGGVENDS 114
AD28s_HUMAN       ELKYKMTINGKIAVLYLKKNKNLLAPGYTETYYNSTGKEITTSPQIMDDCYYQGHILNEK 115
                   :.* :    *:  :* *:*.:.*::  *:** *    *     ***:* : ***:* : ::

AD28_HUMAN        VSDASISTCRGLRGYFSQGDQRYFIEPLSPIHRDGQEHALFKYNPDEKNYDSTCGMDGVL 175
AD28_MOUSE        VSAASISTCQGLRGYISQGDEKYFIEPLSSENLDEQAHALFKDDSNEDQEKSNCGVDDAL 177
AD28_CHICK        DSTASIRACKGLSGYFETRGQKYLIEPLGTS--DRDKHAVY-EFVEDNP-IKTCGVVNSS 173
AD28a_XENTR       GSLASISTCSGLRGLIQTEGQMYLIEPLKDT--DSESHAVY--EAQMET-PKTCGVDNTT 166
AD28b_XENTR       SSLLSISACSGLSGIIQTRGRRYLIEPLKQT--DSEEHAVFPYQAQEST-PVTCGVTNTT 171
AD28bs_XENLA      SSLVSISTCNGLSGIIQTRGRRYLIEPLKQT--DSEEHAVFPYQAQEST-PRTCGVTNTT 171
AD28s_HUMAN       VSDASISTCRGLRGYFSQGDQRYFIEPLSPIHRDGQEHALFKYNPDEKNYDSTCGMDGVL 175
                   *  ** :* ** * :.   .. *:****      * : **::    : .    .**: .

AD28_HUMAN        WAHDLQQNIALPATKLVKLKDRKVQEHEKYIEYYLVLDNGEFKRYNENQDEIRKRVFEMA 235
AD28_MOUSE        WLQGLHQDVALPATRLIKLNDGMVQEPKKYIEYYVVLDNGEFKKYNKNLAEIRKIVLEMA 237
AD28_CHICK        WEVNASSPINSIFKSSNSPEMKAYLKAKKYLEVYVVADNTMYKKYNKDVNTVRQRVFGIV 233
AD28a_XENTR       YREEIVVKKS---RSGSSAEQQQILKAQKYIRLVVVADNSMFIKYNKSITNVKIRIFEVI 223
AD28b_XENTR       YMEGNFSKTS---LATSNAAKVEFLKSKKYIELYMVADKSMFNKYGRSTENVKRRIFEIV 228
AD28bs_XENLA      YMEGNFSKTS---FSTNNEERKEFLKSKKFIQLYMVADNSMFNKYSRSTENVRRRIFEIV 228
AD28s_HUMAN       WAHDLQQNIALPATKLVKLKDRKVQEHEKYIEYYLVLDNGEFKRYNENQDEIRKRVFEMA 235
                  :                .        : :*::.  :* *:  : :*...   ::  :: :

AD28_HUMAN        NYVNMLYKKLNTHVALVGMEIWTDKDKIKITPNASFTLENFSKWRGSVLSRRKRHDIAQL 295
AD28_MOUSE        NYINMLYNKLDAHVALVGVEIWTDGDKIKITPDANTTLENFSKWRGNDLLKRKHHDIAQL 297
AD28_CHICK        NFINTVYKAVNIYVALIGLEIWTDNDKCSLSPIAGSTLDNFSKWRNSDLLKRKRNDNAQL 293
AD28a_XENTR       NFVNVVYKAINTFVALIGIEVWNTTDQFKVVTSASADLAAFADWRKNVLLRRTPNDNAQL 283
AD28b_XENTR       NFVNVVYKQINIFVALTGIEVWDKSDGFTVVSSADEDLKLFSDWRTKNLLPRKAHDNAQF 288
AD28bs_XENLA      NFVNVVYKQMNIFVALTGIEVWDRTDKFTVVSSANEDLRMFSEWRKNDLLPRKAHDNAQF 288
AD28s_HUMAN       NYVNMLYKKLNTHVALVGMEIWTDKDKIKITPNASFTLENFSKWRGSVLSRRKRHDIAQL 295
                  *::* :*: :: .*** *:*:*    *   .: . *.  *  *:.** . *  *. :* **:
                                                                        Δ
AD28_HUMAN        ITATELAGTTVGLAFMSTMCSPY-SVGVVQDHSDNLLRVAGTMAHEMGHNFGMFHD-DYS 353
AD28_MOUSE        ISSTDFSGSTVGLAFMSSMCSPYHSVGIVQDHSNYHLRVAGTMAHEMGHNLGMIHD-YLS 356
AD28_CHICK        ITGHDLEGTTIGLAFIKSICSDVYSAGIIQDHSRNEIAVGATMAHEMGHNLGMSHD-TKD 352
AD28a_XENTR       LTNTDFDGATVGLAYVGTECSETHSAGVIQDHSKASIGVGATVAHELGHNLGMNHD-TDS 342
AD28b_XENTR       ITHIDFDGSTVGLAYVGTMCSSTSSTGVIQDHSQSIAIGATVAHEMGHNLGINHD-EAH 347
AD28bs_XENLA      ITNTDFDGATVGLAYVGTLCSSTLSTGVIQDHSQQSISIGATVAHEMGHNLGMNHDEEPH 348
AD28s_HUMAN       ITATELAGTTVGLAFMSTMCSPY-SVGVVQDHSDNLLRVAGTMAHEMGHNFGMFHD-DYS 353
                  ::  :: *:*:***:: : **    *.*::****    : :..*:***:***:*: **

AD28_HUMAN        CKCPSTICVMDKALSFYIPTDFSSCSRLSYDKFFEDKLSNCLFNAPLPTDIISTPICGNQ 413
AD28_MOUSE        CKCPSEVCVMEQSLRFHMPTDFSSCSRVNYKQFLEEKLSHCLFNSPLPSDIISTPVCGNQ 416
AD28_CHICK        CMCHSRVCIMTDTVSSIIPKKFSSCSLQDFEKYMLNDMPKCLTNIPDINAIIAPSSCGNG 412
AD28a_XENTR       CSCSASSCIMAPYLSVNTPRLFSSCSLQNYQDFLLSRMPQCMLAVPLKEDIIAPAVCGNK 402
AD28b_XENTR       CTCSTGSCIMEPALSFNTPREFSLCSHQNYQDFILQKMPLCMKDVPKKTEIQTPPVCGNK 407
AD28bs_XENLA      CTCSSGSCIMEPSLSFNTPREFSLCSHQNYQDFILQKMPLCMTDKPQKTEIQTPPLCGNK 408
AD28s_HUMAN       CKCPSTICVMDKALSFYIPTDFSSCSRLSYDKFFEDKLSNCLFNAPLPTDIISTPICGNQ 413
                  * * :  *:*   :    *  ** **  .:..:: . :. *:   *     * :.. ***

AD28_HUMAN        LVEMGEDCDCGTSEECTNICCDAKTCKIKATFQCALGECCEKCQFKKAGMVCRPAKDECD 473
AD28_MOUSE        LLEMNEDCDCGTPKECTNKCCDARTCKIKAGFQCALGECCEKCQLKKPGVVCRAAKDECD 476
AD28_CHICK        FVEKGEECDCGTSEECTNACCDPETCKLTAGSMCAQGDCCEDCQYKRAGAVCREAKDDCD 472
AD28a_XENTR       FTEIGEDCDCGTVEECTNQCCDAATCKLKSTAKCAEGDCCDNCQIKKAGSVCRAAKDDCD 462
AD28b_XENTR       FTELGEECDCGSISECTNPCCNATTCKLKAGAQCAEGQCCDSCKWTKAGTVCRSKRHDCD 467
AD28bs_XENLA      FTELGEECDCGTVEECTNPCCDAFTCKLKSEAQCAEGQCCSKCQWTKAGTVCRDSKGDCD 468
AD28s_HUMAN       LVEMGEDCDCGTSEECTNICCDAKTCKIKATFQCALGECCEKCQFKKAGMVCRPAKDECD 473
                  : * .*:****: .**** **:. ***:.:   ** *:**..*: .:.* ***  : :**
```

```
AD28_HUMAN        LPEMCNGKSGNCPDDRFQVNGFPCHHGKGHCLMGTCPTLQEQCTELWGPGTEVADKSCYN 533
AD28_MOUSE        LPEVCDGKSSHCPGDRFRVNGSPCQNGHGYCLKGKCPTLQQQCMDMWGPGTKVANTSCYK 536
AD28_CHICK        LPEMCTGYSGNCPSDRFRMNGHPCNNGEGFCYMGICPTRESQCRAAFGPQATGGAASCYR 532
AD28a_XENTR       LADMCDGKSSVCPSDRFRVNGFPCSNGKGYCFNGKCPTHQSQCTTLWGASSVPSEDSCFN 522
AD28b_XENTR       LAEMCDGQSAECPSDRFRVNGFPCRNGEGYCYNGLCPTLQGLCSALWGPSSVVADDSCFN 527
AD28bs_XENLA      LTEMCDGQSAECPSDRFRVNGFPCINGEGYCYNGICPTLQGMCSVLWGPDSVVADDSCFN 528
AD28s_HUMAN       LPEMCNGKSGNCPDDRFQVNGFPCHHGKGHCLMGTCPTLQEQCTELWGPGRRTNPFPCAC 533
                  *.::* * *. **.***::** ** :*.*.*  * *** :  *   :*.       .*

AD28_HUMAN        RNEGGSKYGYCRRVDDTLIPCKANDTMCGKLFCQGGSDNLPWKGRIVTFLTCKTFDPEDT 593
AD28_MOUSE        QNEGGTKYGYCHVENGTHMPCKAKDAMCGKLFCEGGSGDLPWKGLTISFLTCKLFDPEDT 596
AD28_CHICK        MNEKGVYYGYCRKERGSHVPCKKKDIMCGKLYCSGGWEMP-SYGSLVTFESCKASFPRNG 591
AD28a_XENTR       VNTRGVDYGYCTMAGATYVPCKPKDVKCGMLFCYGGSSQPSIYAAVAEFSRCRAVLAQG- 581
AD28b_XENTR       YNLRGANNGYCQDSKGNQVPCKQSDVKCGVLYCSGGYSTP-NLGGYYIRGECKTTLYPT- 585
AD28bs_XENLA      YNLRGLSYAFCLDSRGNNIPCKPRDIKCGTLHCSGGSERP-ISGGYYTIGECKTTWSPT- 586
AD28s_HUMAN       AKENHFR----------------------------------------------------- 540
                   :

AD28_HUMAN        SQEIGMVANGTKCGDNKVCINAECVDIEKAYKSTNCSSKCKGHAVCDHELQCQCEEGWIP 653
AD28_MOUSE        SQGVDMVANGTKCGTNKVCINAECVDMEKTYKSANCSSKCKGHAVCDHELQCQCKEGWAP 656
AD28_CHICK        DTDLGMILNGTKCGDGMVCSNGECVYAEDVFRSSDCSAKCPGHAVCDHEMQCQCEEGWAP 651
AD28a_XENTR       ----GMVQNGTKCGDGMVCYNGACSSIESAYRSTNCSAQCSGHAVCDHEMLCQCEEGWAP 637
AD28b_XENTR       ----FLVENGTKCGDNMVCFMGECTSIQTAFGPSDCDAKCPEHAVCDHENQCRCEEGWAP 641
AD28bs_XENLA      ----FIALNGTKCGENMR-------SPDQKYCSSNCNRK--------------------- 614
AD28s_HUMAN       ------------------------------------------------------------

                               xxxxxxxxxxxxxxxxxxx
AD28_HUMAN        PDCDDSSVVFHFSIVVGVLFPMAVIFVVVAMVIRHQSSREKQKKDQRPLSTTGTRPHKQK 713
AD28_MOUSE        PDCENSATVFHFSIVVGVLFPLAVIFVVVAIVIQRQSARRKQRRVQRLPSTKDAKLHNQK 716
AD28_CHICK        PNCDSSSAVTSFAVIAGVLAVLTVIV-IAAVLLFRFRVFKKSSHTRRGPGATNQVFVDQE 710
AD28a_XENTR       PTCDVASPTNIIIIVVVIVIALALVIGLVFLARFYRGKGKKQSSSSFPATVTVEGSTNPS 697
AD28b_XENTR       PDCANFTGTNTIIVVVAIICVIVLFI--LLFVWFKWSSRRKQRTSRTRVTGAVNPAFNVR 699
AD28bs_XENLA      ----------------------------------KCGSKR-QNLSVHRLM---------- 629
AD28s_HUMAN       ------------------------------------------------------------

AD28_HUMAN        RKPQMVKAVQPQE-------------MSQMK-PHVYDLPVEGNEPPASFHKDTNALPP-- 757
AD28_MOUSE        CRPQKVKDVQPQE-------------MSQMKKLHVSDLPSEEPEPPPDVLITKPNFPPPP 763
AD28_CHICK        QRPREHPGLAVPTQKINDKKLLLPVPPLQENKPQLRSPVIRPKGPPPPVPCTKPAFSHTQ 770
AD28a_XENTR       FRNQPPPQMQMPLPQVHK--------PKPATDKSWPPSRVGYQAPQYSVTTASPQAEP-K 748
AD28b_XENTR       EKPWGPAGSNAATPQLDS--------RFPHNPPKPPAQSQKPLATHPPTGRSWPQTQVGY 751
AD28bs_XENLA      ------------------------------------------------------------
AD28s_HUMAN       ------------------------------------------------------------

AD28_HUMAN        -TVFKDNPMSTPKDSNPEA--------------- 775
AD28_MOUSE        IPVSLTGRAKVPFVKTPHPFSQQIGRVYLK---- 793
AD28_CHICK        DMFAPEKKPACLPVPKGKPPPPPKALKPPVNPRV 804
AD28a_XENTR       LKKPTVAPPPVPPAKPAPPPAPPKPLKPPVRN-- 780
AD28b_XENTR       PVRPVNAPPPLPTTKPAYPRAPPQAWTPNYRN-- 783
AD28bs_XENLA      ----------------------------------
AD28s_HUMAN       ----------------------------------
```

Additional File 3. Complete sequence alignment of ADAM28 from representative vertebrate species. Sequences of human, mouse, chicken and *Xenopus* ADAM28 proteins were aligned using ClustalX. Soluble forms of *X. laevis* and human ADAM28 (AD28bs_XENLA and AD28s_HUMAN, respectively) are also included. The conserved zinc-binding motif (indicated with “Δ”) and methionine-turn in the catalytic center are highlighted in grey. Arrow points to signal peptide cleavage sites, and residues in the transmembrane region are indicated with “x”.
