# Supplementary material for: Conservation and divergence of ADAM family proteins in the Xenopus genome
Source: BMC Evol Biol. 2010 Jul 14;10:211. doi: 10.1186/1471-2148-10-211 (PMC3055250; doi:10.1186/1471-2148-10-211)
Supplement: Additional file 4 — Complete sequence alignment of ADAM9 from representative vertebrate species. [file 1471-2148-10-211-S4.PDF]

```
                                        Signal peptidase
                                                 ▼
AD9_HUMAN         ---MGSGARFPSGTLRVRWLLLLGLVGPVLGAARPGFQQTSHLSSYEIITPWRLTRERRE 57
AD9_MOUSE         ---MGPRALSPLASLRLRWLLACGLLGPVLEAGRPDLEQTVHLSSYEIITPWRLTRERRE 57
AD9_CHICKEN       --------------------LVVAILFCFLG-----FQEISQLSSYEVIIPQKLGRERRE 35
AD9_XENTR         -----MAATYPSHPFSIGICLV-AICTLIQG-----IEQTSFLSSYNIIIPQKVARARRD 49
AD9_XENLA         MRGHPMASTCPSQPFSIGICLISAICTLIQG-----IEQTSFLSSYNIIIPQKVARARRD 55
AD9_DANRE         ---------MTRHVNTFVLILINFSICPVQA-----IDQTSYLKKYDVVKPQLVQMRWRR 46
                                      *       .       :::   *..*::: *  :    *

AD9_HUMAN         AP-RPYSKQ-------VSYVIQAEGKEHIIHLERNKDLLPEDFVVYTYNKEGTLITDHPN 109
AD9_MOUSE         AL-GPSSQQ-------ISYVIQAQGKQHIIHLERNTDLLPNDFVVYTYDKEGSLLSDHPN 109
AD9_CHICKEN       TSNASSTQDK------VSYAIEIEGKEYIFHVEKNKDLLPKDFTVYTYNKEGKLQSEYPD 89
AD9_XENTR         TA-GSSGENS------LSYAIEVEGKKHVLHLEKNRDFIASDFAVYTYAANGSIVSSQLQ 102
AD9_XENLA         TS-GSSGENS------LSYAIEVEGKKHVLHLEKNRDFIARDFAVYTYAENGSIISSQLQ 108
AD9_DANRE         YADPSHKQPEERHADIITYSVRIEGSDRILHVTKNTDFLSKNFVVISHKTP-KKGKVQPE 105
                      .  :        ::* :. :*.. ::*: :* *::. :*.* ::    .  .   :

AD9_HUMAN         IQNHCHYRGYVEGVHNSSIALSDCFGLRGLLHLENASYGIEPLQNSSHFEHIIYRMDDVY 169
AD9_MOUSE         VQSHCHYRGYVEGVQNSAVAVSACFGLRGLLHLENASFGIEPLHNSSHFEHIFYPMDGIH 169
AD9_CHICKEN       VQDHCYYQGYVEGILDSVVAVSTCSGLRGLVTIGNVTYGIEPMDSSSGSEHIVYSLDNVK 149
AD9_XENTR         DKEHCHYQGYAEGVPGSVAAISTCSGLRGMLHLQNSSYGIEPLDSSDKFQHLIYRLEDVK 162
AD9_XENLA         DKEHCHYQGYAEGVPESVVAISTCSGLRGMLHLQNSSYGIEPLDNSDKFQHLVYRMEDVK 168
AD9_DANRE         RMVQCYYQGHVEGYEDSLVALSTCEGIRGVIIIGNKSYGLEPVLHSKANEHLLFLLEDSH 165
                     :*:*:*:.**   *  *:* * *:**:: : * ::*:**:  *.  :*:.: ::.

AD9_HUMAN         KEPLKCGVSNKDIEKETAKDEEEEPPSMTQLLRRRRAVLPQTRYVELFIVVDKERYDMMG 229
AD9_MOUSE         QEPLRCGVSNRDTEKEGTQGDEEEHPSVTQLLRRRRAVLPQTRYVELFIVVDKERYDMMG 229
AD9_CHICKEN       KEPSMCGVMTEGHEEG-EHTGENHHPSMTQLLRKKRAILHQTRYVELFIVVDKEKFEDFG 208
AD9_XENTR         SEPMVCGVTAADKEPE----TDINPLSMTQLLRRKRAVLHQTRYVELFLVVDKERFDMLG 218
AD9_XENLA         SEPMVCGVTAADKEPE----TELNPPSMTQLLRRKRAILPQTRYVELFIVVDKERFDMLS 224
AD9_DANRE         SEPFVCGLENETSLSE----DHSRYADMSMFLRKKR-TLPQTKYVELALVVDQKRFIIKN 220
                  .**  **:             .  .  .:: :**::*  * **:**** :***::::   .

AD9_HUMAN         RNQTAVREEMILLANYLDSMYIMLNIRIVLVGLEIWTNGNLINIVGGAGDVLGNFVQWRE 289
AD9_MOUSE         RNQTAVREEMIRLANYLDSMYIMLNIRIVLVGLEIWTDRNPINIIGGAGDVLGNFVQWRE 289
AD9_CHICKEN       KSETEVREHMVQLANFLDSMYIMLNIHIVLVGLEIWKYENIISTDGGAGDVLANFVQWRE 268
AD9_XENTR         RNETAVRAEMVQLSNYLDSMYTMLNIRIVLVGLMIWTDGNKIGIDGTAGDVLGRFVQWRE 278
AD9_XENLA         RNESAVRAEMVQLSNYLDSMYTMLNIRIVLVGLMIWTDSNKIAIDGTAGDVLGRFVQWRE 284
AD9_DANRE         RSVNAVRDEMVQLANLLDTYFKQLNIRIALIGLKIFDTGNPFSVDGNAGDVLGRFVDWRK 280
                  :. . ** .*: *:* **: :  ***:*.*:** *:   * :   * *****..**:**:

AD9_HUMAN         KFLITRRRHDSAQLVLKKG--FGGTAGMAFVGTVCSRSHAGGINVFGQITVETFASIVAH 347
AD9_MOUSE         KFLITRRRHDSAQLVLKKG--FGGTAGMAFVGTVCSRSHAGGINVFGQITVETFASIVAH 347
AD9_CHICKEN       KNLVLRRRHDSAQFVLKKG--FGGTAGMAYVGTVCSKSHAGGINVFGKISIQMFASIMAH 326
AD9_XENTR         TSLVSLRRHDSAQLVLKKG--FGGTAGMAFVGTVCSRSHAGGINVFSHYNVQSFASIVAH 336
AD9_XENLA         TNLVSLRRHDSAQLVLKKG--FGGTAGMAFVGTVCSRSHAGGINVFSHYNVQSFASIVAH 342
AD9_DANRE         TTLSPQIRNDAAQLVVGQGAYPGGVLGMAFVGSVCSASTSGAISVFSDNNLQYYSTVAAH 340
                  . *    *:*:**:*: :*   **. ***:**:*** * :*.*.**.. .:: :::: **
                      Δ
AD9_HUMAN         ELGHNLGMNHDDGRDCSCGAKSCIMNSGASGSRNFSSCSAEDFEKLTLNKGGNCLLNIPK 407
AD9_MOUSE         ELGHNLGMNHDDGRECFCGAKSCIMNSGASGSRNFSSCSAEDFEKLTLNKGGSCLLNIPK 407
AD9_CHICKEN       ELGHNLGMNHDDERVCHCGASSCIMSSGASGSRNFSSCSAEDFEKLTLNKGGSCLLNVPR 386
AD9_XENTR         ELGHNLGMNHDDGRNCLCSVDTCIMNSGATGSKNFSSCSEEDFEKLTLSKGGTCLLNMPK 396
AD9_XENLA         ELGHNLGMNHDDGRNCFCSVDNCIMNSGATGSKNFSSCSEEDFEKLTLNKGGTCLLNVPK 402
AD9_DANRE         ELGHNLGMSHDSNG-CSC---QCIMAPSASGSTKFSDCSDNAFERLIQGGGGACLRNIPA 396
                  ********.**.   * *    *** ..*:** :**.** : **:*  . ** ** *:*

AD9_HUMAN         PDEAYSAPSCGNKLVDAGEECDCGTPKECELDPCCEGSTCKLKSFAECAYGDCCKDCRFL 467
AD9_MOUSE         PDEAYSAPSCGNKLVDPGEECDCGTAKECEVDPCCEGSTCKLKSFAECAYGDCCKDCQFL 467
AD9_CHICKEN       PDETYSIPYCGNKLVDMGEECDCGSPKECENDPCCEPGTCRLRPTAECAYGDCCKNCRIL 446
AD9_XENTR         PDEAYSAPFCGNKLVDIGEECDCGSPKECEKDPCCEPGTCKLRSGAQCAYGTCCQNCRFS 456
AD9_XENLA         PDEAYSAPFCGNKLVDVGEECDCGNPKECEKDPCCEPGTCKLRSGAQCAYGTCCQNCGFS 462
AD9_DANRE         QDSIISVPRCGNGILESGEECDCGTPQECN-TTCCNAATCTFTKGSTCAAGSCCQKCQII 455
                   *.  * * *** ::: *******..:**:  .**: .** :   : ** * **:.* :

AD9_HUMAN         PGGTLCRGKTSECDVPEYCNGSSQFCQPDVFIQNGYPCQNNKAYCYNGMCQYYDAQCQVI 527
AD9_MOUSE         PGGSMCRGKTSECDVPEYCNGSSQFCPPDVFIQNGYPCQNSKAYCYNGMCQYYDAQCQVI 527
AD9_CHICKEN       PGGTECRASNNECDLPEYCNGTSQFCQPDFTVQNGHPCHNEEAYCYNGVCQYYDAQCQDI 506
AD9_XENTR         SGGTVCRAVANECDLPEYCNGSSPFCQPDVYIQNGHPCQNSKAYCYNGMCQYYDAQCQAI 516
AD9_XENLA         PGGTVCRAVANECDLPEYCNGSSPFCQPDVYIQNGNPCQNSKAYCYNGMCQSYDAQCQAI 522
AD9_DANRE         VAGTPCRPSINPCDLPEYCGGESPYCPSDFYMMDGLPCNNNAAYCFEGRCQTFDYQCKQI 515
                   .*: **   . **:****.* * :* .*. : :* **:*. ***::* ** :* **: *
```

```
AD9_HUMAN       FGSKAKAAPKDCFIEVNSKGDRFGNCGFSGNEYKKCATGNALCGKLQCENVQEIPVFGIV 587
AD9_MOUSE       FGSKAKAAPRDCFIEVNSKGDRFGNCGFSGSEYKKCATGNALCGKLQCENVQDMPVFGIV 587
AD9_CHICKEN     FGSKAKAAPNICFAKVNSKGDRFGNCGFHGHDYKKCSSWNAMCGKLQCENVETMPVFGIK 566
AD9_XENTR       FGSKAKSAPPVCYQEVNSKGDRFGNCGFQGSDYRKCDTRNARCGKLQCENVETMPVFGIR 576
AD9_XENLA       FGSKAKSAPPICYQEVNSKGDRFGNCGFQGNDYRKCDTRNARCGKLQCENVESMPVFGIR 582
AD9_DANRE       FGSGATKADDKCFTNVNTYGNAFGNCGYSGTFPKPCSVQNAMCGKLQCLFNSNNPPLGAT 575
                *** *. *   *: :**: *: *****: *   : *   ** ******   .  * :*

AD9_HUMAN       PAIIQTPSRG--TKCWGVDFQLGSDVPDPGMVNEGTKCGAGKICRNFQCVDASVLN--YD 643
AD9_MOUSE       PAIIQTPSRG--TKCWGVDFQLGSDVPDPGMVNEGTKCDAGKICRNFQCVNASVLN--YD 643
AD9_CHICKEN     PAIIRTPSDG--TTCWGVDFQLGSDVPDPGMVNEGTKCDNGKVCRHFQCVSASVLN--YD 622
AD9_XENTR       PSYIQTPIHGTTTICWGVDFQLGSDVPDPAMVNEGTKCDEGKICSKFQCVDASVLQ--YD 634
AD9_XENLA       PSYIQTPIHGTSTVCWGVDFQLGSDVPDPAMVNEGTKCDESKVCSKFQCVDASVLQ--YD 640
AD9_DANRE       VSVQKIEGGT--ITCMNADFNMGPDVPDPAYVKTASVCAPGKLCANFTCFNSSVMDQSRK 633
                 :  :         * ..**::*.*****. *: .: *  .*:* :* *..:**::   .

                                                                      xxxxxx
AD9_HUMAN       CDVQKKCHGHGVCNSNKNCHCENGWAPPNCETKGYGGSVDSGPTYNEMNTALRDGLLVFF 703
AD9_MOUSE       CDIQGKCHGHGVCNSNKNCHCEDGWAPPHCDTKGYGGSVDSGPTYNAKSTALRDGLLVFF 703
AD9_CHICKEN     CDVEKQCHGHGVCNNNRNCHCEPGWAPPFCNTKGYGGSIDSGPPYNDKDNSLRNGLLVFF 682
AD9_XENTR       CDVQKKCGGNGVCNTNKNCHCNEGWAFPNCQTKGYGGSIDSGPTYNDKDTSLRDGLLVFF 694
AD9_XENLA       CDVQKKCGGNGVCNSNKNCHCNEGWAFPNCQTEGYGGSIDSGPTYNDKDTSLRDGLLVFF 700
AD9_DANRE       CDAQRDCSSNGVCNDRYHCHCNNGWGPPNCNKGGRGGSIDSGP--AEIDYSLRNGLLIFF 691
                ** : .* .:**** . :***: **. * *:. * ***:****     . :**:***:**

                xxxxxxxxxxxxxxx
AD9_HUMAN       FLIVPLIVCAIFIFIKRDQLWRSYFRKKRSQTYESDGKNQANPSRQPG------------ 751
AD9_MOUSE       FLIVPLVAAAIFLFIKRDELRKT-FRKKRSQ--MSDGRNQANVSRQPGD----------- 749
AD9_CHICKEN     FLVLPLLIAAALAFARRDQLKRW-FRRLMSRCHSVGAENFIFTFMSLQSPPPRT------ 735
AD9_XENTR       FLIVPLLALGAFVFFRRNELKRRFCRKKRSQAHEVDNKNQTGGERQASGPPRNT--AAPG 752
AD9_XENLA       FLIVPLLGLGAFVFFRRNELKRRFCRKKRSQAHEVDNKNETGGERLASGTPRNAPSTAPG 760
AD9_DANRE       LLVVPILVVLIIILLY--VFKRDSLKRFLKGCPSIRKRPTNAANGSSNTP---------- 739
                :*::*::    : :     : :   ::  .       .

AD9_HUMAN       ----SVPR-----HVSP------VTPP--------------------------------- 763
AD9_MOUSE       ---PSISRPPGGPNVSRPPGGPGVSRPPGGP--------------------GVSRPPGGP 786
AD9_CHICKEN     ---EAEPRDFPRRGVPHGMPYPSRGVPMSETEAVFPKCVQNLLSILSTDDMEPNTFPVPS 792
AD9_XENTR       RGVPNPGPPRNAHNVPPNRGTQPTGPPRNMPNPAQNQGPS--GPPRNVPPAGPYRSTPAT 810
AD9_XENLA       RGVPNAGPPRNALNVPPNRGTPVTGPPRNMPNPAQSQRPSALGPPRNVPPAGPYRSMPAT 820
AD9_DANRE       ------------SNVQANGNTTRASAPQSMT----------------------------- 758
                              *           *

AD9_HUMAN       ---REVPIYANR---FAVPTYAAKQPQQFPSRPP-PPQPKVSSQGNLIPARPAPAPPLYS 816
AD9_MOUSE       GVSRPPPGHGNR---FPVPTYAAKQPAQFPSRPP-PPQPKISSQGNLIPARPAPAPPLYS 842
AD9_CHICKEN     YPINQHPQQAYRQSYYPSPQYQALQPQKLPARPP-PPQQKSVPQGHYFPSRPAPLPPK-- 849
AD9_XENTR       SPPHSTPYQPNN---FAVPTYTVKQPQHTPSRPP-LPHQREAA-ANITPSRPAPAPPL-- 863
AD9_XENLA       SPPHNTPYQPNN---FAVPTYTVKQPQHTPSRPP-LPHQREAA-ANIIPSRPAPAPPL-- 873
AD9_DANRE       --EIQTAHQSQP----TDPKYQQDTTVNQPRQGPGVPKP--------IPPRQTQVPV--- 801
                      .         . * *    . : * : *  *:          *.* :  *

AD9_HUMAN       SLT 819
AD9_MOUSE       SLT 845
AD9_CHICKEN     ---
AD9_XENTR       ---
AD9_XENLA       ---
AD9_DANRE       ---
```

Additional File 4. Complete sequence alignment of ADAM9 from representative vertebrae species. Sequences of human, mouse, chicken (partial without N-terminus), *X. tropicalis*, *X. laevis* and zebrafish ADAM15 proteins were aligned using ClustalX. The zinc-binding motif (indicated with “Δ”) and methionine-turn in the catalytic center, along with the conserved cytoplasmic proline residues, are highlighted in grey. Arrow points to signal peptide cleavage sites, and residues in the transmembrane region are indicated with “x”.
