# Supplementary material for: Conservation and divergence of ADAM family proteins in the Xenopus genome
Source: BMC Evol Biol. 2010 Jul 14;10:211. doi: 10.1186/1471-2148-10-211 (PMC3055250; doi:10.1186/1471-2148-10-211)
Supplement: Additional file 5 — Complete sequence alignment of ADAM11 from representative vertebrate species. [file 1471-2148-10-211-S5.PDF]

```
                                        Signal peptidase
                                        ▼
AD11_Human      MRLLRRWAFAALLLS-LLPTPGLGTQGPAGALRWGGLPQLGGP----GAP---EVTEPSR 52
AD11_Mouse      MRRLRRWAIAALLLLPLLPPPGLGALGPRGALHWRSSAHVGSP----ESPEGSEVTEPSR 56
AD11_XENTR      MMCLGLFWVVAAVISANAAMHPLGSS-LVNTIVPPITAHSDSPKRWYQKLDHTQITHPSR 59
AD11_DANRE      MLAKRCLLLWAAVIARLAVT-AWGSSTKRDGAEWGR-----------ENAYTVEHALPQR 48
                *       . * ::         *:    .                       : : *.*

AD11_Human      LVRESSGGEVRKQ-QLDTRVRQEPPGGPPVHLAQVSFVIPAFNSNFTLDLELNHHLLSSQ 111
AD11_Mouse      LVRQSSGGEVRKP-QLDTRVRQDPPRGTPVHLAQVSFVIPAFDSNFTLDLELNHHLLSSQ 115
AD11_XENTR      LVGHTSGAETHRY-QLNTRVRSENP----------------------------------- 83
AD11_DANRE      LLQRTDAEEELPHGHLGTLTKTDGDGTHPIHLAQITFLVKAFGIPFVLDLELNHDLLSSN 108
                *: .:.. *     :*.* .: :

AD11_Human      YVERHFSREGTTQHSTGAGDHCYYQGKLRGNPHSFAALSTCQGLHGVFSDGNLTYIVEPQ 171
AD11_Mouse      YVERHFSREGTRQHSTGAGDHCYYHGKLRGNPQSFAALSTCQGLHGVFSDGNLTYIVEPK 175
AD11_XENTR      ------------------------NDQTEKWPHAIERIKKCT------------------ 101
AD11_DANRE      YVERHFEKDGQSFQTLG-GEHCYYHGHVRGVPRSWAALSTCHGLQGMFSDGNFSYGIEPL 167
                                        :.: .  *::   :..*

AD11_Human      EVAGPWGAPQGPLPHLIYRTPLLPDPLGCR-------EPGCLFAVPAQSAPPNRPRLRRK 224
AD11_Mouse      EIAGPWGPPQGPLPHLIYRTPLLPAPLGCR-------EPGCLFAVPAQSALPNWPKLRRK 228
AD11_XENTR      -------------------------------------EPDCELHTIIPGS-----KIRRK 119
AD11_DANRE      HNSSDQDANIHVVYRMADIRLMPHFSGSTRNSSDSDINNDYPVSMETSQLELTDGLRRAK 227
                                                     : .  .              * *

AD11_Human      RQVRRGHPTVHSETKYVELIVINDHQLFEQMRQSVVLTSNFAKSVVNLADVIYKEQLNTR 284
AD11_Mouse      RQVRRGHPTVHSETKYVELIVINDHQLFEQMRQSVVLTSNFAKSVVNLADVIYKEQLNTR 288
AD11_XENTR      RQVRRTSHSALTETKYVELMVVNDRYLFDQQRQSVVLTSSFAKSVVNLADVIFREQLNTR 179
AD11_DANRE      RQVRRGPRTVQSETKYVELLVVNDYDLFVQMRRSSPQTRNFAKAVVNMADAIYKEQLNTR 287
                *****   :. :*******:*:**  ** * *:*   * .***:***:**.*::******

AD11_Human      IVLVAMETWADGDKIQVQDDLLETLARLMVYRREGLPEPSDATHLFSGR--TFQSTSSGA 342
AD11_Mouse      IVLVAMETWADGDKIQVQDDLLETLARLMVYRREGLPEPSDATHLFSGR--TFQSTSSGA 346
AD11_XENTR      IVLVGMETWTSTDKITGSEDPLQVLDEFMRYRRAEILDHSDTTHLFSGRTFTFKSSRSGA 239
AD11_DANRE      IVLVAMETWSTQNMVSVGDDPLVTLRDFMKYRKENIKEKSDTAHLLSGR--TFQSSRSGT 345
                ****.****:  : :   :* * .*  :* **:  : : **::**:***  **:*: **:

AD11_Human      AYVGGICSLSHGGGVNEYGNMGAMAVTLAQTLGQNLGMMWNKHRSSAGDCKCPDIWLGCI 402
AD11_Mouse      AYVGGICSLSRGGGVNEYGNMGAMAVTLAQTLGQNLGMMWNKHRSSAGDCKCPDIWLGCI 406
AD11_XENTR      AYFGGICSPSHGGGVNEYGNIGVMAVTLAQTLGQNLGMMWNKLRTTAGDCKCPDLWLGCI 299
AD11_DANRE      AYIEGICSPTRGGGVNEYGNVGPMAITLCQSLGQNLGMMWNKDRATAGDCRCPDPWLGCI 405
                **. **** ::*********:* **:**.*:*********** *::****:*** *****

AD11_Human      MEDTGFYLPRKFSRCSIDEYNQFLQEGGGSCLFNKPLKLLDPPECGNGFVEAGEECDCGS 462
AD11_Mouse      MEDTGFYLPRKFSRCSIDEYNQFLQEGGGSCLFNKPLKLLDPPECGNGFVEAGEECDCGS 466
AD11_XENTR      MEDTGYYLPQKFSRCSVDEYSQFLQDGGGSCLFNKPLKLLDPPSCGNGFVELGEECDCGS 359
AD11_DANRE      MEDTGYYLPRKFSRCSIEEYIQFLQQGGGSCLFNKPNKLLDPPECGNGFVEQGEECDCGS 465
                *****:***:******::** ****:********** ******.******* ********

AD11_Human      VQECSRAG-GNCCKKCTLTHDAMCSDGLCCRRCKYEPRGVSCREAVNECDIAETCTGDSS 521
AD11_Mouse      VQECSRAG-GNCCKKCTLTHDAMCSDGLCCRRCKYEPRGVSCREAVNECDIAETCTGDSS 525
AD11_XENTR      PAECNKSGAGNCCKKCTLSHDAMCSDGLCCRGCKYEPRGTVCRGSLNECDVPETCPGDSS 419
AD11_DANRE      QVDCSRAG-GACCKKCTLTHDAMCSNGLCCNRCKYEQRGVICRDAVNDCDVPETCSGDSS 524
                  :*.::* * *******:******:****. **** **. ** ::*:**:.***.****

AD11_Human      QCPPNLHKLDGYYCDHEQGRCYGGRCKTRDRQCQVLWGHAAADRFCYEKLNVEGTERGSC 581
AD11_Mouse      QCPPNLHKLDGYYCDHEQGRCYGGRCKTRDRQCQALWGHAAADRFCYEKLNVEGTERGNC 585
AD11_XENTR      VCPANLHKQDGYFCDNEQGRCFGGRCKTRDRQCHALWGRSASDRFCYEKLNIEGTEKGNC 479
AD11_DANRE      KCPHNVHKLDGYMCDAGLGRCYGGRCKTRDAQCQALWGHNAADRMCYEKLNIEGTERGNC 584
                 ** *:** *** **   ***:******** **:.***: *:**:******:****:*.*

AD11_Human      GRKGS--GWVQCSKQDVLCGFLLCVNISGAPRLGDLVGDISSVTFYHQGKELDCRGGHVQ 639
AD11_Mouse      GRKGS--GWVQCSKQDVLCGFLLCVNISGAPRLGDLGGDISSVTFYHQGKELDCRGGHVQ 643
AD11_XENTR      GRDRQ--NWIQCSKQDVLCGYLLCSNISGIPQIGELNGDITSMSFYHQNRYLDCRGGQVM 537
AD11_DANRE      GQDSSSHNWIQCNKQDVLCGFLLCTNITVKPRYGDLHGESTSLTIYHQNKYLDCRGGHAV 644
                *:. .  .*:**.*******:*** **:  *: *:* *: :*:::***.: ******:.

AD11_Human      LADGSDLSYVEDGTACGPNMLCLDHRCLPASAFNFSTCPGSGERRICSHHGVCSNEGKCI 699
AD11_Mouse      LADGSDLSYVEDGTACGPNMLCLDHRCLPASAFNFSTCPGSGERRICSHHGVCSNEGKCI 703
AD11_XENTR      LPDGSCLGYVEDGTPCGPNMMCLERRCLPASAFNFSTCPGSWNGVICSDHGVCSNEGKCI 597
AD11_DANRE      LEDGTDLGYVEDGTPCGPNMMCLDHRCLPVTTFNLSSCPGSSFSLVCSDHGTCSNEVKCI 704
                * **: *.******.*****:**::****.::**:*:****    :**.**.**** ***
```

```
                                                   xxxxxxxxxxxxxxxxxxxxx
AD11_Human      CQPDWTGKDCSIHNPLPTSPPTGETERYKGPSGTNIIIGSIAGAVLVAAIVLGGTGWGFK 759
AD11_Mouse      CQPDWTGKDCSIHNPLPTSPPTGETERYKGPSGTNIIIGSIAGAVLVAAIVLGGTGWGFK 763
AD11_XENTR      CHPEWTGKDCSVYDPLPIPKPTGVVEKYKGPSGTNIIIGSIAGAVLIAAIVLGGTGWGFK 657
AD11_DANRE      CDSDYTGKDCSVYDPIPDPQPPDGPEKYKGPSGTNIIIGSIAGAILLAAIVLGGTGWGFK 764
                *..::******:::*:* . *..  *:*****************:*:*************

AD11_Human      NIRRGRSG----GA- 769
AD11_Mouse      NIRRGRYDPTQQGAV 778
AD11_XENTR      NIRRGRSG----GG- 667
AD11_DANRE      NIRRGRSG----GG- 774
                ****** .    *.
```

Additional File 5. Complete sequence alignment of ADAM11 from representative vertebrae species. Sequences of human, mouse, *X. tropicalis*, and zebrafish ADAM15 proteins were aligned using ClustalX. Arrows point to signal peptide cleavage sites, and residues in the transmembrane region are indicated with “x”.
