# Supplementary material for: Conservation and divergence of ADAM family proteins in the Xenopus genome
Source: BMC Evol Biol. 2010 Jul 14;10:211. doi: 10.1186/1471-2148-10-211 (PMC3055250; doi:10.1186/1471-2148-10-211)
Supplement: Additional file 6 — Phylogenetic trees generated using alternative models. [file 1471-2148-10-211-S6.PDF]

A

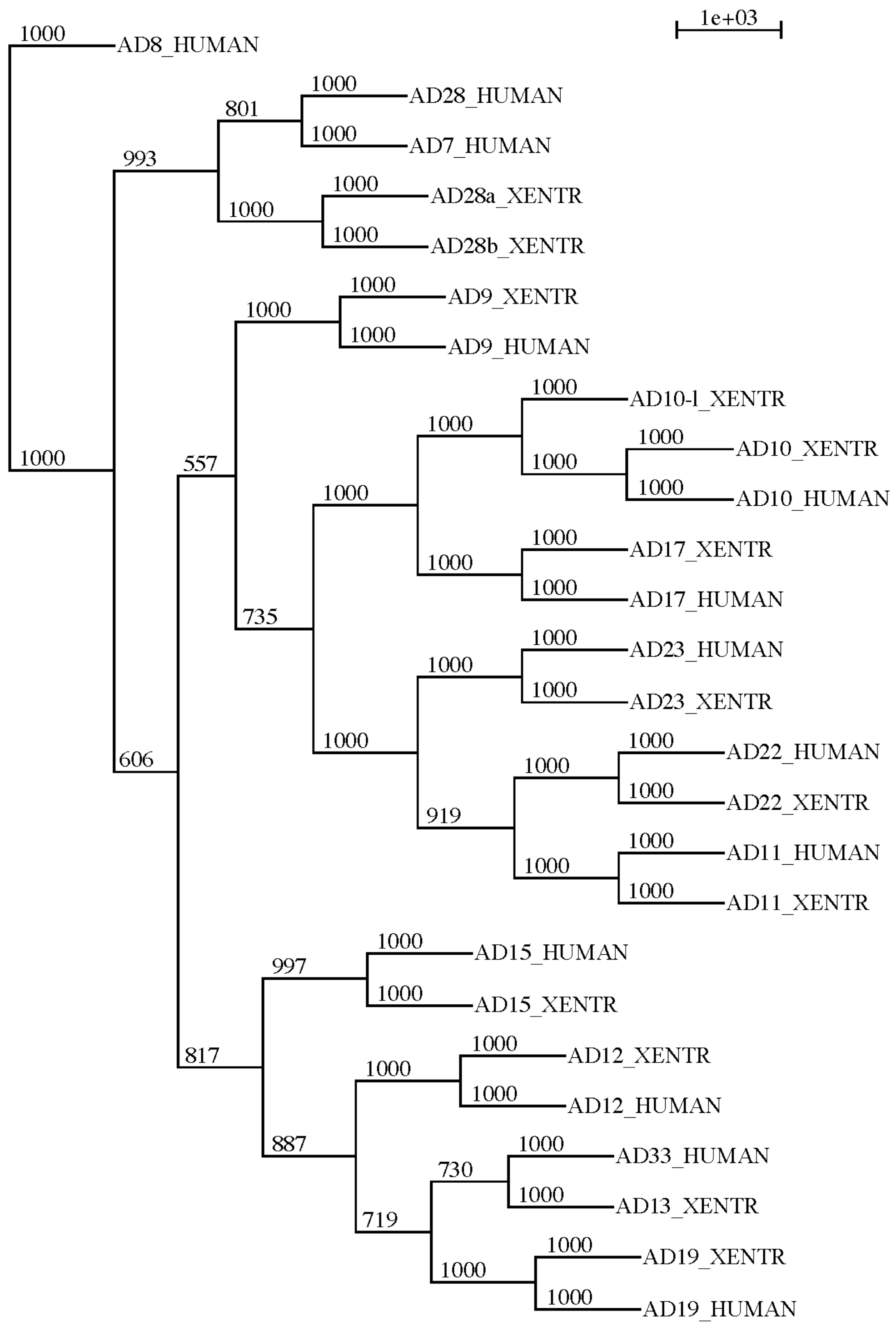
1e+03
AD8_HUMAN
AD28_HUMAN
AD7_HUMAN
AD28a_XENTR
AD28b_XENTR
AD9_XENTR
AD9_HUMAN
AD10-l_XENTR
AD10_XENTR
AD10_HUMAN
AD17_XENTR
AD17_HUMAN
AD23_HUMAN
AD23_XENTR
AD22_HUMAN
AD22_XENTR
AD11_HUMAN
AD11_XENTR
AD15_HUMAN
AD15_XENTR
AD12_XENTR
AD12_HUMAN
AD33_HUMAN
AD13_XENTR
AD19_XENTR
AD19_HUMAN
1000
993
801
557
735
606
919
997
817
887
730
719

B

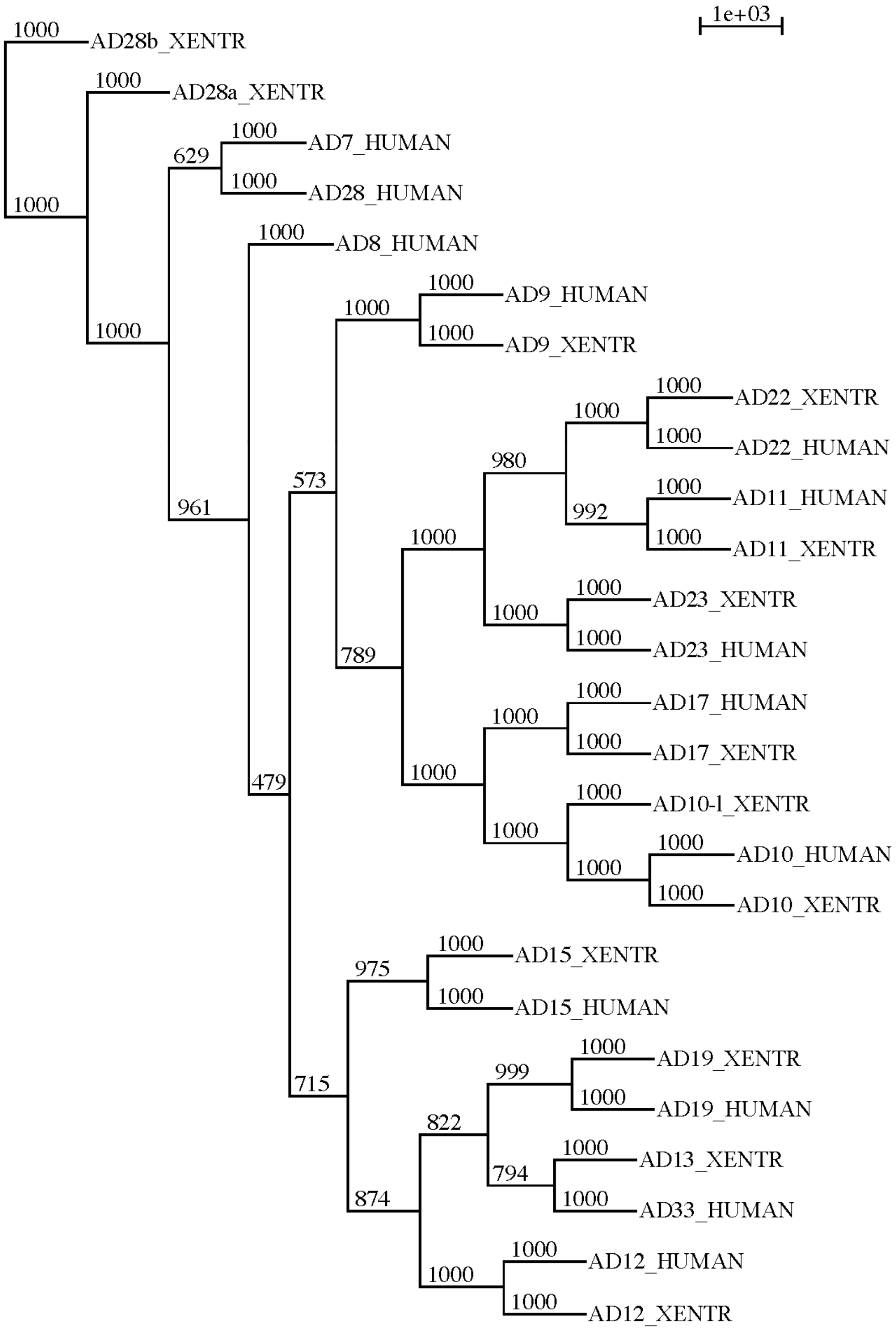
1e+03
AD28b_XENTR
AD28a_XENTR
AD7_HUMAN
AD28_HUMAN
AD8_HUMAN
AD9_HUMAN
AD9_XENTR
AD22_XENTR
AD22_HUMAN
AD11_HUMAN
AD11_XENTR
AD23_XENTR
AD23_HUMAN
AD17_HUMAN
AD17_XENTR
AD10-1_XENTR
AD10_HUMAN
AD10_XENTR
AD15_XENTR
AD15_HUMAN
AD19_XENTR
AD19_HUMAN
AD13_XENTR
AD33_HUMAN
AD12_HUMAN
AD12_XENTR
629
961
479
573
789
980
992
975
715
874
822
999
794

C

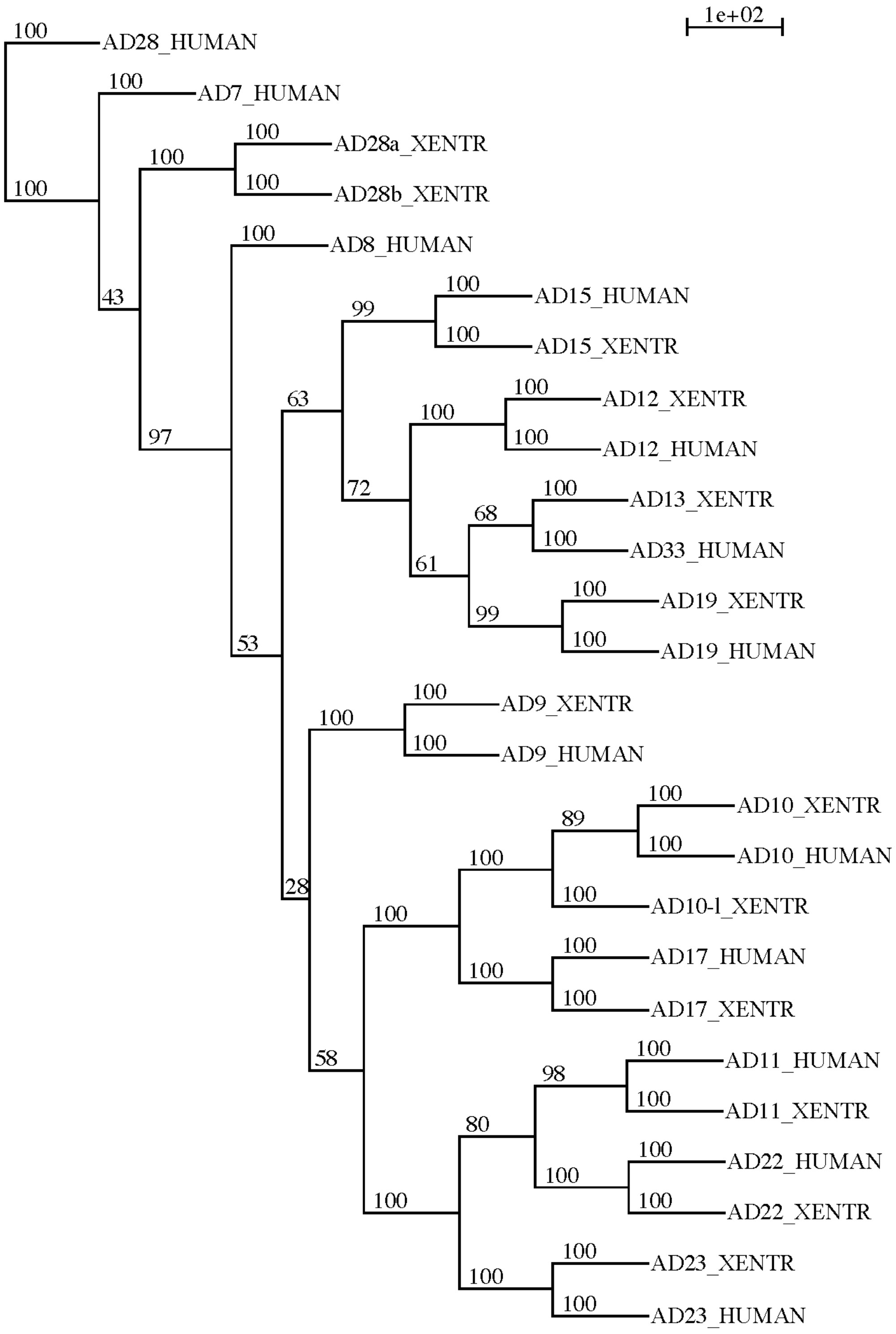
1e+02
AD28_HUMAN
AD7_HUMAN
AD28a_XENTR
AD28b_XENTR
AD8_HUMAN
AD15_HUMAN
AD15_XENTR
AD12_XENTR
AD12_HUMAN
AD13_XENTR
AD33_HUMAN
AD19_XENTR
AD19_HUMAN
AD9_XENTR
AD9_HUMAN
AD10_XENTR
AD10_HUMAN
AD10-l_XENTR
AD17_HUMAN
AD17_XENTR
AD11_HUMAN
AD11_XENTR
AD22_HUMAN
AD22_XENTR
AD23_XENTR
AD23_HUMAN

D

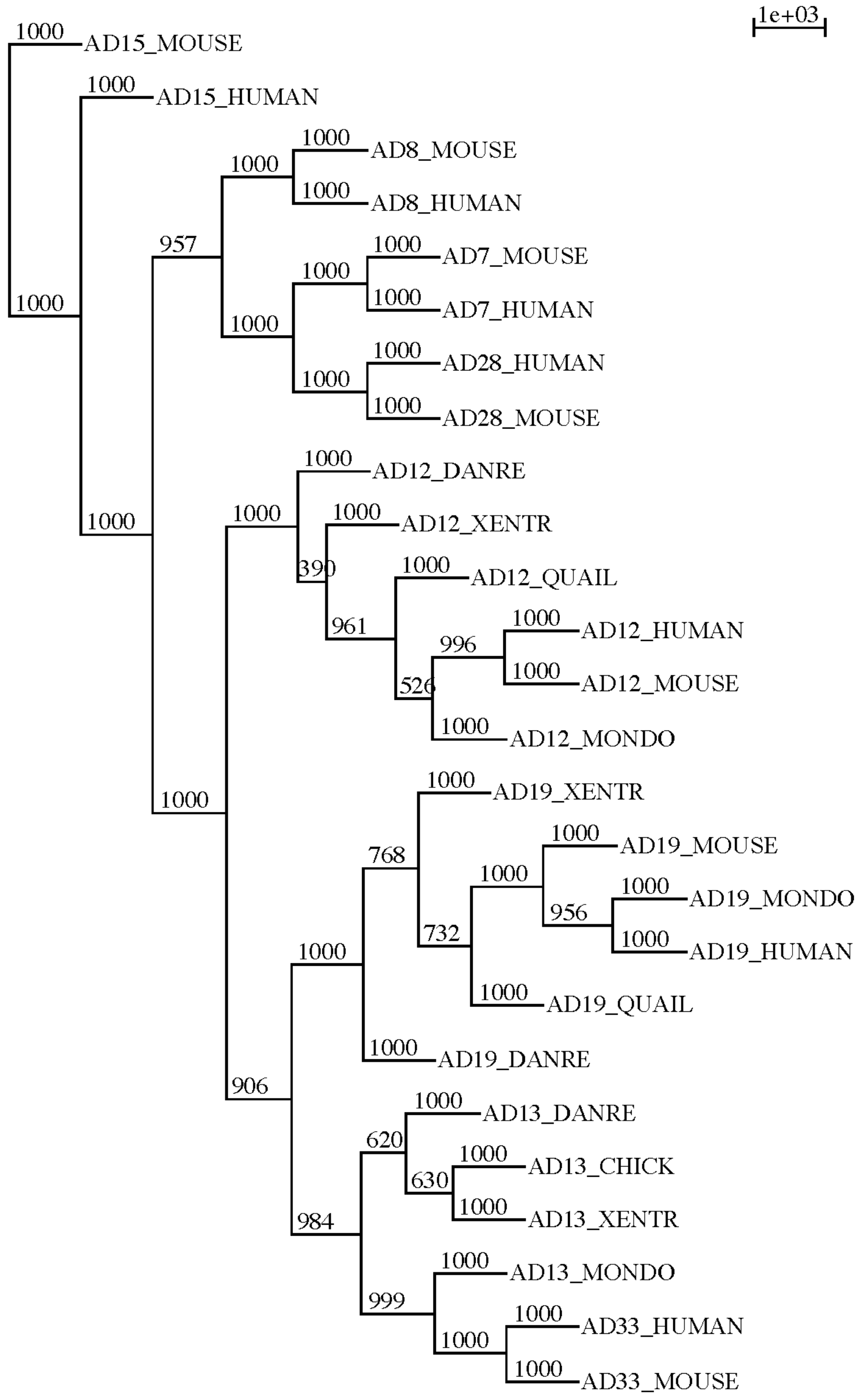
1e+03
AD15_MOUSE
AD15_HUMAN
AD8_MOUSE
AD8_HUMAN
AD7_MOUSE
AD7_HUMAN
AD28_HUMAN
AD28_MOUSE
AD12_DANRE
AD12_XENTR
AD12_QUAIL
AD12_HUMAN
AD12_MOUSE
AD12_MONDO
AD19_XENTR
AD19_MOUSE
AD19_MONDO
AD19_HUMAN
AD19_QUAIL
AD19_DANRE
AD13_DANRE
AD13_CHICK
AD13_XENTR
AD13_MONDO
AD33_HUMAN
AD33_MOUSE

E

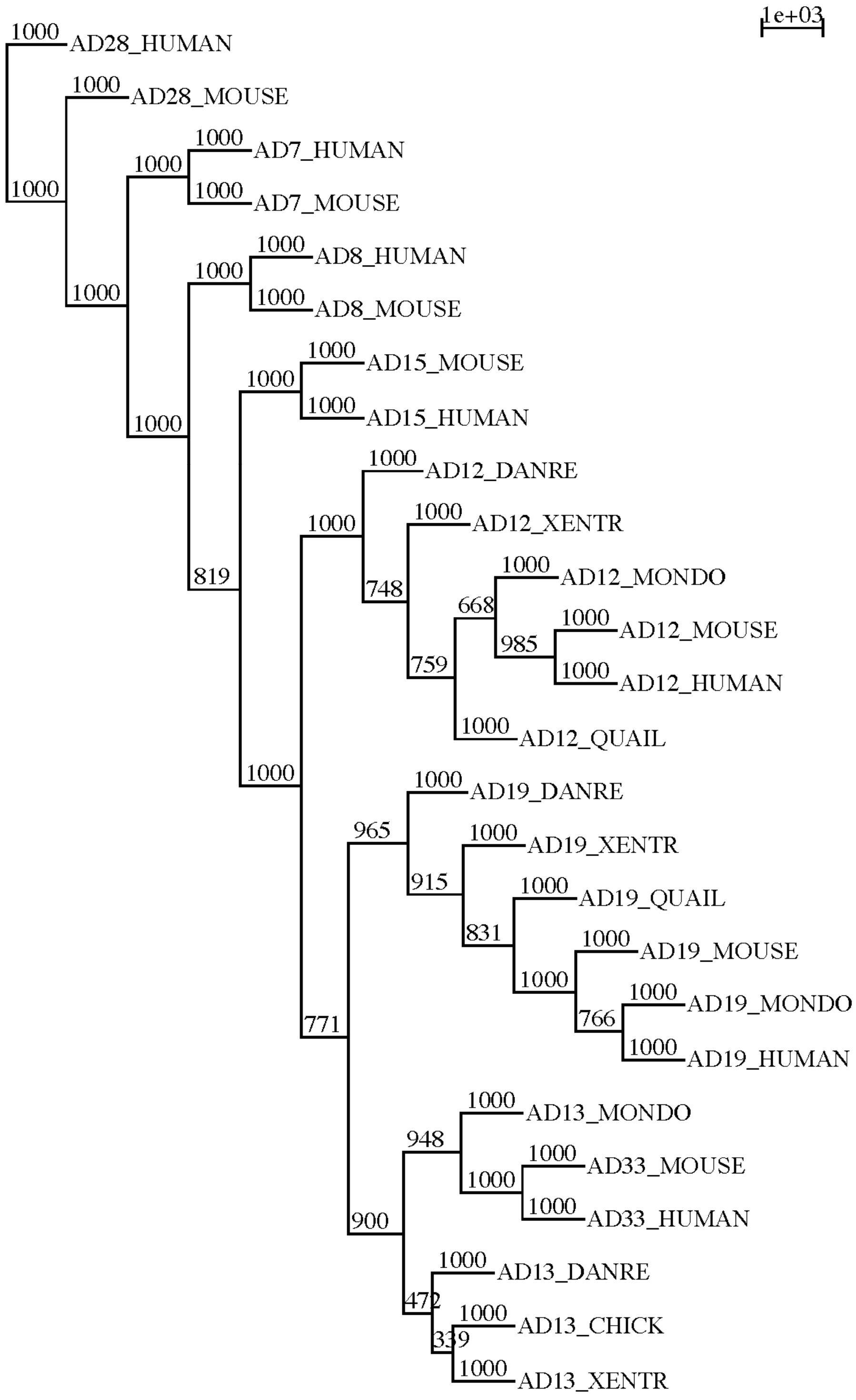
1e+03
AD28_HUMAN
AD28_MOUSE
AD7_HUMAN
AD7_MOUSE
AD8_HUMAN
AD8_MOUSE
AD15_MOUSE
AD15_HUMAN
AD12_DANRE
AD12_XENTR
AD12_MONDO
AD12_MOUSE
AD12_HUMAN
AD12_QUAIL
AD19_DANRE
AD19_XENTR
AD19_QUAIL
AD19_MOUSE
AD19_MONDO
AD19_HUMAN
AD13_MONDO
AD33_MOUSE
AD33_HUMAN
AD13_DANRE
AD13_CHICK
AD13_XENTR
1000
819
748
668
985
759
965
915
831
766
771
948
900
472
339

F

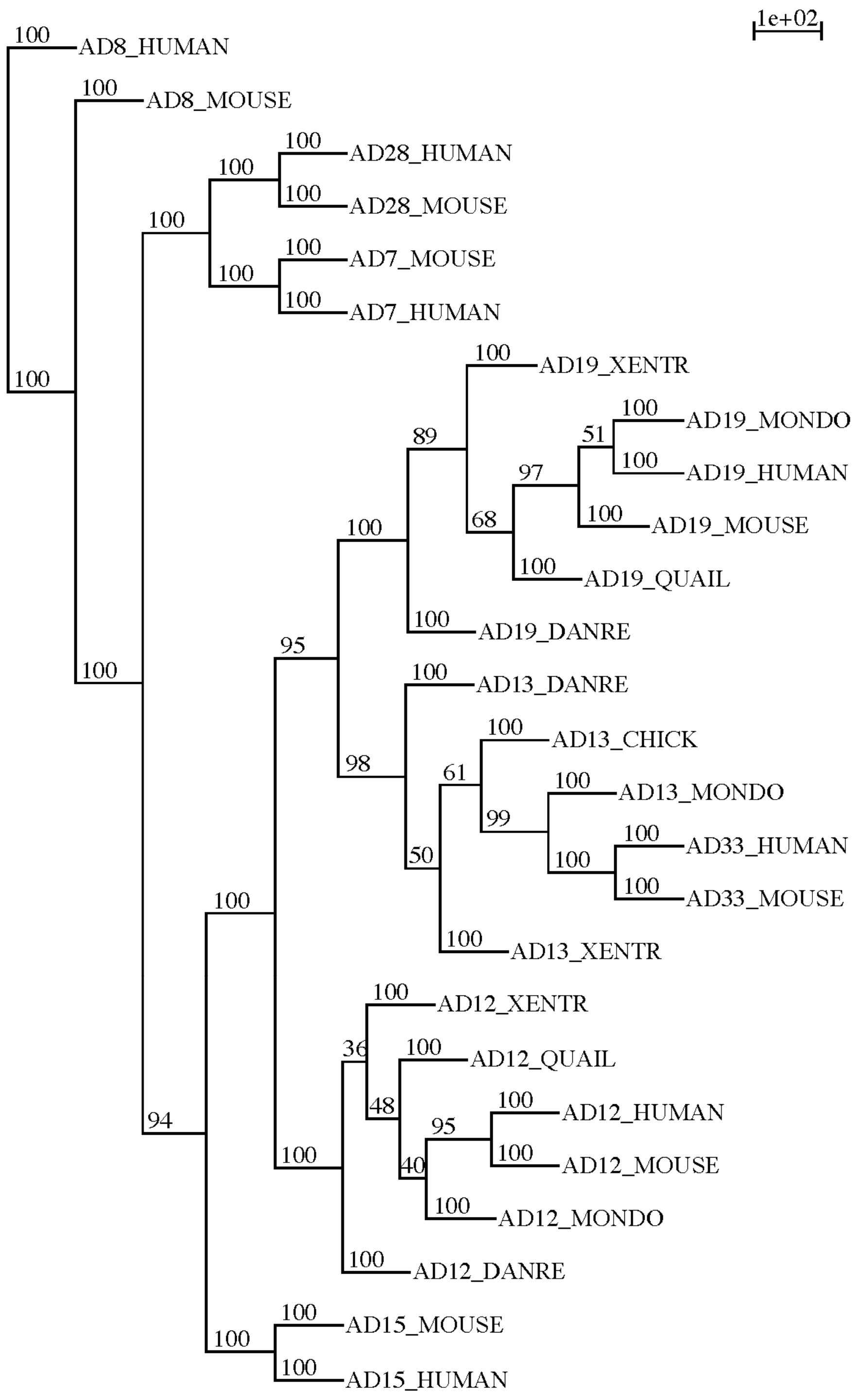
1e+02
AD8_HUMAN
AD8_MOUSE
AD28_HUMAN
AD28_MOUSE
AD7_MOUSE
AD7_HUMAN
AD19_XENTR
AD19_MONDO
AD19_HUMAN
AD19_MOUSE
AD19_QUAIL
AD19_DANRE
AD13_DANRE
AD13_CHICK
AD13_MONDO
AD33_HUMAN
AD33_MOUSE
AD13_XENTR
AD12_XENTR
AD12_QUAIL
AD12_HUMAN
AD12_MOUSE
AD12_MONDO
AD12_DANRE
AD15_MOUSE
AD15_HUMAN

G

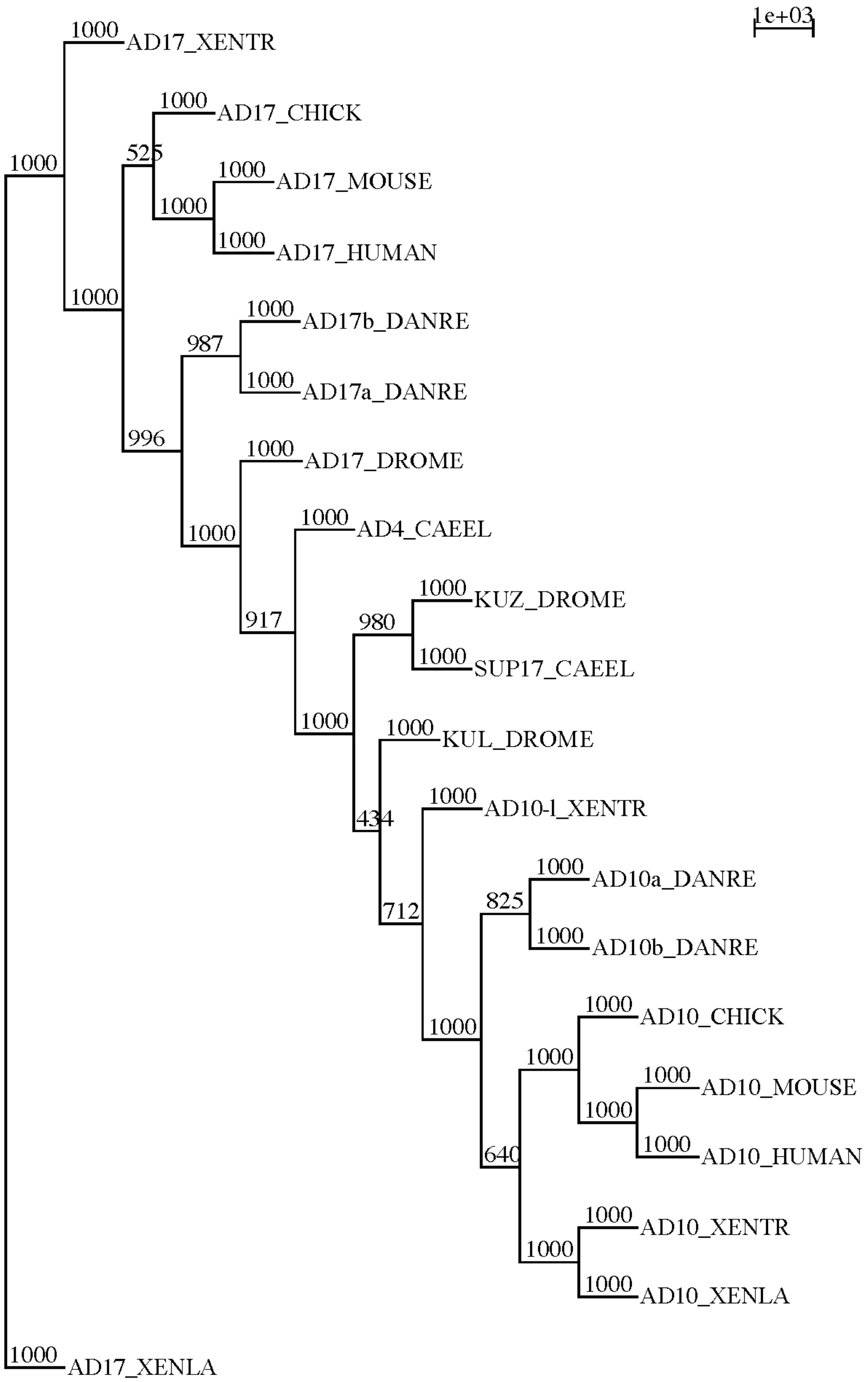
1e+03
AD17_XENTR
AD17_CHICK
AD17_MOUSE
AD17_HUMAN
AD17b_DANRE
AD17a_DANRE
AD17_DROME
AD4_CAEEL
KUZ_DROME
SUP17_CAEEL
KUL_DROME
AD10-l_XENTR
AD10a_DANRE
AD10b_DANRE
AD10_CHICK
AD10_MOUSE
AD10_HUMAN
AD10_XENTR
AD10_XENLA
AD17_XENLA
525
987
996
917
980
434
712
825
640

H

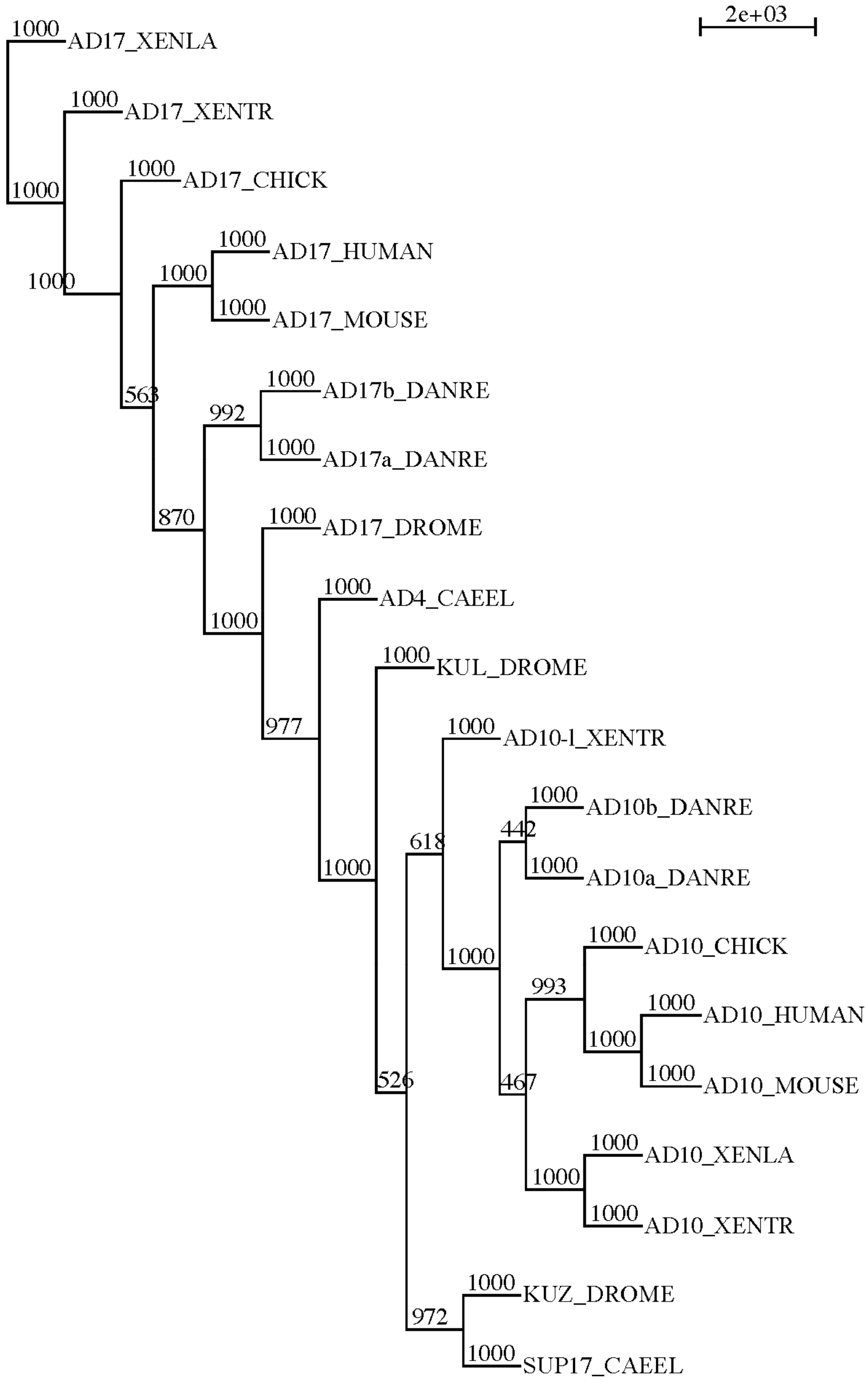

2e+03
AD17_XENLA
AD17_XENTR
AD17_CHICK
AD17_HUMAN
AD17_MOUSE
AD17b_DANRE
AD17a_DANRE
AD17_DROME
AD4_CAEEL
KUL_DROME
AD10-1_XENTR
AD10b_DANRE
AD10a_DANRE
AD10_CHICK
AD10_HUMAN
AD10_MOUSE
AD10_XENLA
AD10_XENTR
KUZ_DROME
SUP17_CAEEL

I

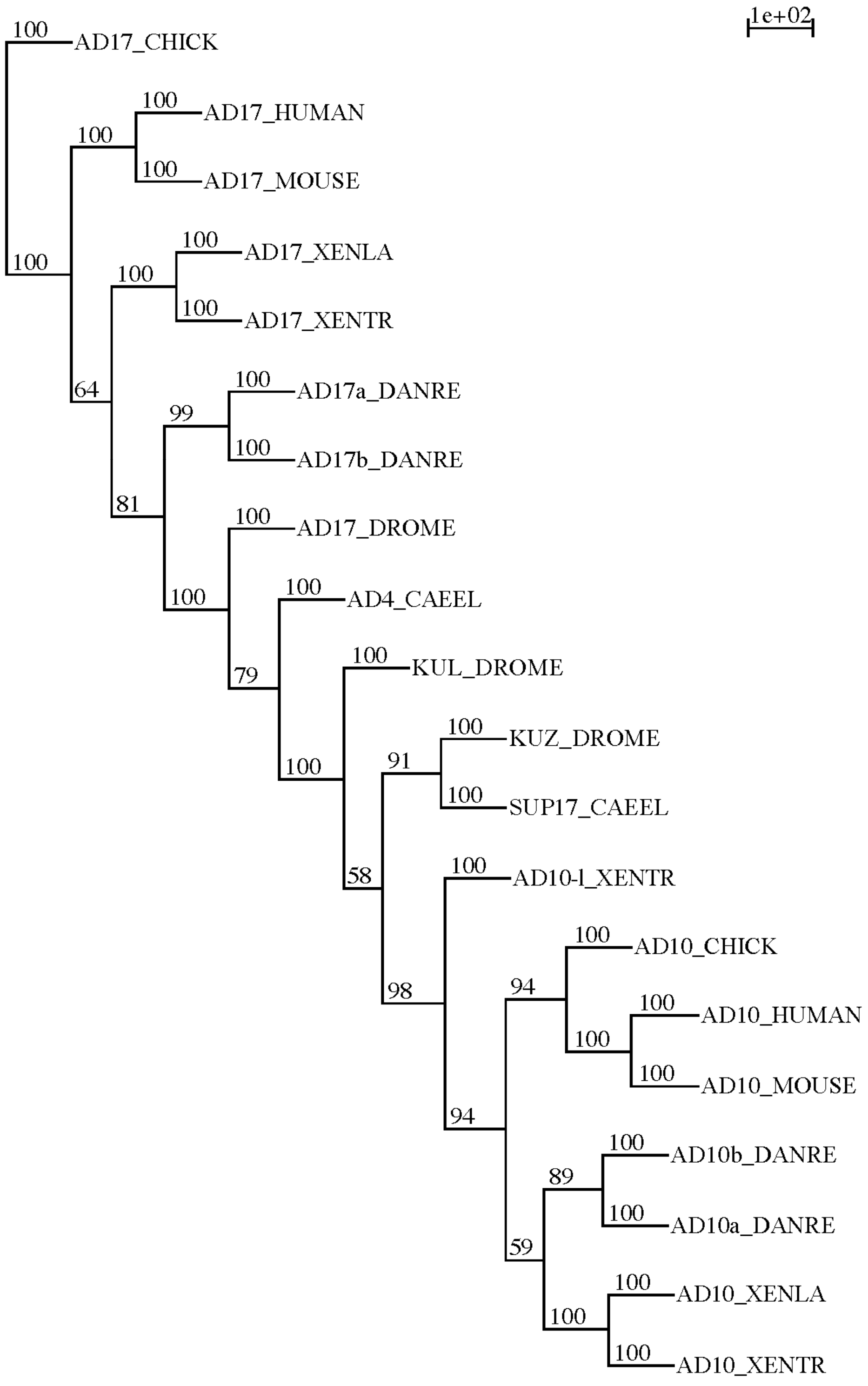
1e+02
AD17_CHICK
AD17_HUMAN
AD17_MOUSE
AD17_XENLA
AD17_XENTR
AD17a_DANRE
AD17b_DANRE
AD17_DROME
AD4_CAEEL
KUL_DROME
KUZ_DROME
SUP17_CAEEL
AD10-l_XENTR
AD10_CHICK
AD10_HUMAN
AD10_MOUSE
AD10b_DANRE
AD10a_DANRE
AD10_XENLA
AD10_XENTR
100
64
81
99
79
58
91
98
94
59
89

Additional File 6. Phylogenetic trees generated using alternative models. Trees shown in Figs. 1A, 3A and 8, respectively, were rebuilt using Phylip Neighbor (A, D, G), Protpars (B, E, H), and Proml (C, F, I) analyses. Bootstrap values (out of 1000 for Phylip Neighbor and Protpars, and out of 100 for Proml) are displayed on the branches.
