# Supplementary material for: Conservation and divergence of ADAM family proteins in the Xenopus genome
Source: BMC Evol Biol. 2010 Jul 14;10:211. doi: 10.1186/1471-2148-10-211 (PMC3055250; doi:10.1186/1471-2148-10-211)
Supplement: Additional file 7 — Sequences of primers used in RT-PCR experiments shown in Figure 3B. [file 1471-2148-10-211-S7.PDF]

Additional File 7. Sequences of primers used in RT-PCR experiments shown in Fig. 3B

Primer name	Sequence
adam12 forward	AATGGAAGACTGTGAAGTGGCG
adam12 reverse	TTGTCCTGGCTGGCACCAAATGTG
adam13 forward	CAGCAGCAGTGTATCCATCTTTGG
adam13 reverse	CCCAGGTTTGTCACAGTAAGGAGG
adam19 forward	ATGCGATTGGTGTAGCAGCC
adam19 reverse	GCACTGTTGTTGGTGGGTAAGG
β-actin forward	CCAACAGCAGTAGTTCCTTCCGTAG
β-actin reverse	CGTCATAAATAGCAACAGTGTGGG
